# Cardiomyocyte-specific overexpression of syndecan-4 in mice results in activation of calcineurin-NFAT signalling and exacerbated cardiac hypertrophy

**DOI:** 10.1007/s11033-022-07985-y

**Published:** 2022-10-07

**Authors:** Ida G. Lunde, J. Magnus Aronsen, A. Olav Melleby, Mari E. Strand, Jonas Skogestad, Bård A. Bendiksen, M. Shakil Ahmed, Ivar Sjaastad, Håvard Attramadal, Cathrine R. Carlson, Geir Christensen

**Affiliations:** 1 Institute for Experimental Medical Research, Oslo University Hospital and University of Oslo, Oslo, Norway; 2 KG Jebsen Center for Cardiac Research, University of Oslo, Oslo, Norway; 3 Division of Diagnostics and Technology, Akershus University Hospital, Lørenskog, Norway; 4 Institute for Medical Biosciences, University of Oslo, Oslo, Norway; 5 Institute for Surgical Research, Oslo University Hospital and University of Oslo, Oslo, Norway; 6 Institute for Experimental Medical Research (IEMR), Oslo University Hospital Ullevaal, Building 7, 4th floor, Kirkeveien 166, 0407 Oslo, Norway

**Keywords:** Heart failure, Fibrosis, Inflammation, Proteoglycan, Matrix, Calcium

## Abstract

**Background:**

Cardiomyocyte hypertrophy is a hallmark of cardiac dysfunction in patients with aortic stenosis (AS), and can be triggered by left ventricular (LV) pressure overload in mice by aortic banding (AB). Syndecan-4 is a transmembrane heparan sulphate proteoglycan which is found increased in the myocardium of AS patients and AB mice. The role of syndecan-4 in cardiomyocyte hypertrophy is not well understood.

**Purpose of the study:**

We developed mice with cardiomyocyte-specific overexpression of syndecan-4 (*Sdc4-Tg*) and subjected these to AB to examine the role of syndecan-4 in hypertrophy and activation of the pro-hypertrophic calcineurin-NFAT signalling pathway.

**Methods and results:**

*Sdc4-Tg* mice showed exacerbated cardiac remodelling upon AB compared to wild type (WT). At 2–6 weeks post-AB, *Sdc4-Tg* and WT mice showed similar hypertrophic growth, while at 20 weeks post-AB, exacerbated hypertrophy and dysfunction were evident in *Sdc4-Tg* mice. After cross-breeding of *Sdc4-Tg* mice with NFAT-luciferase reporter mice, we found increased NFAT activation in *Sdc4-Tg* hearts after AB. Immunoprecipitation showed that calcineurin bound to syndecan-4 in *Sdc4-Tg* hearts. Isolated cardiomyocytes from *Sdc4-Tg* mice showed alterations in Ca^2+^ fluxes, suggesting that syndecan-4 regulated Ca^2+^ levels, and thereby, activating the syndecan-4-calcineurin complex resulting in NFAT activation and hypertrophic growth. Similarly, primary cardiomyocyte cultures from neonatal rats showed increased calcineurin-NFAT-dependent hypertrophic growth upon viral *Sdc4* overexpression.

**Conclusion:**

Our study of mice with cardiomyocyte-specific overexpression of *Sdc4* have revealed that syndecan-4 is important for activation of the Ca^2+^-dependent calcineurin-NFAT signalling pathway, hypertrophic remodelling and dysfunction in cardiomyocytes in response to pressure overload.

**Supplementary Information:**

The online version contains supplementary material available at 10.1007/s11033-022-07985-y.

## Introduction

Heart failure carries high morbidity, mortality and societal costs [1–3]. Cardiac hypertrophy, inflammation and fibrosis are central remodelling processes leading to cardiac dysfunction and failure [4], and can be triggered by left ventricular (LV) pressure overload in patients with hypertension or aortic stenosis (AS).

Proteoglycans, proteins substituted with covalently attached glycosaminoglycan (GAG) chains [5], are believed to play important roles during cardiac remodelling and failure [6–9]. Syndecan-4 is a transmembrane heparan sulphate proteoglycan consisting of an ectodomain with GAG chains extending into the extracellular matrix (ECM), a transmembrane domain, and a short cytoplasmic tail involved in signalling and binding to the cytoskeleton [5, 9–17]. The GAG-substituted ectodomain can be shed from the cell surface [18]. Syndecan-4 levels are elevated in hearts of AS patients and mice subjected to aortic banding (AB) [13, 19–23]. We have shown that the Ca^2+^-activated phosphatase calcineurin binds directly to the cytoplasmic domain of syndecan-4 [13], activating downstream nuclear factor of activated T-cell (NFAT) transcription factors, i.e., a central pathway directing pathological cardiomyocyte remodelling [24–27]. These data suggest that syndecan-4 regulates hypertrophy through calcineurin-NFAT signalling.

Syndecan-4 localizes to the costamere and Z-disc of cardiomyocytes [28], areas linking the cytoskeleton to the ECM that are believed to be important for sensing mechanical stress. Cardiomyocyte syndecan-4 expression is increased in response to mechanical stress and inflammatory stimuli [21]. Studies of constitutive syndecan-4 knock-out (*Sdc4-KO*) mice suggest that syndecan-4 regulates cardiac hypertrophy [13, 19–22], however, its specific role in cardiomyocyte remodelling has been difficult to tease out. Syndecan-4 is expressed at comparable levels in cardiac myocytes and fibroblasts [19], and while it is clear that syndecan-4 is important for fibroblast function, formation of focal adhesions, fibrosis and immune cell infiltration [7–9, 21, 29], its role in cardiomyocytes remains incompletely understood.

Here, we developed a mouse line with cardiomyocyte-specific overexpression of syndecan-4 (*Sdc4-Tg*) and subjected these to AB to examine the role of syndecan-4 in cardiomyocytes specifically.

## Results

### Generation of mice with cardiomyocyte-specific overexpression of syndecan-4

**Fig. 1:**
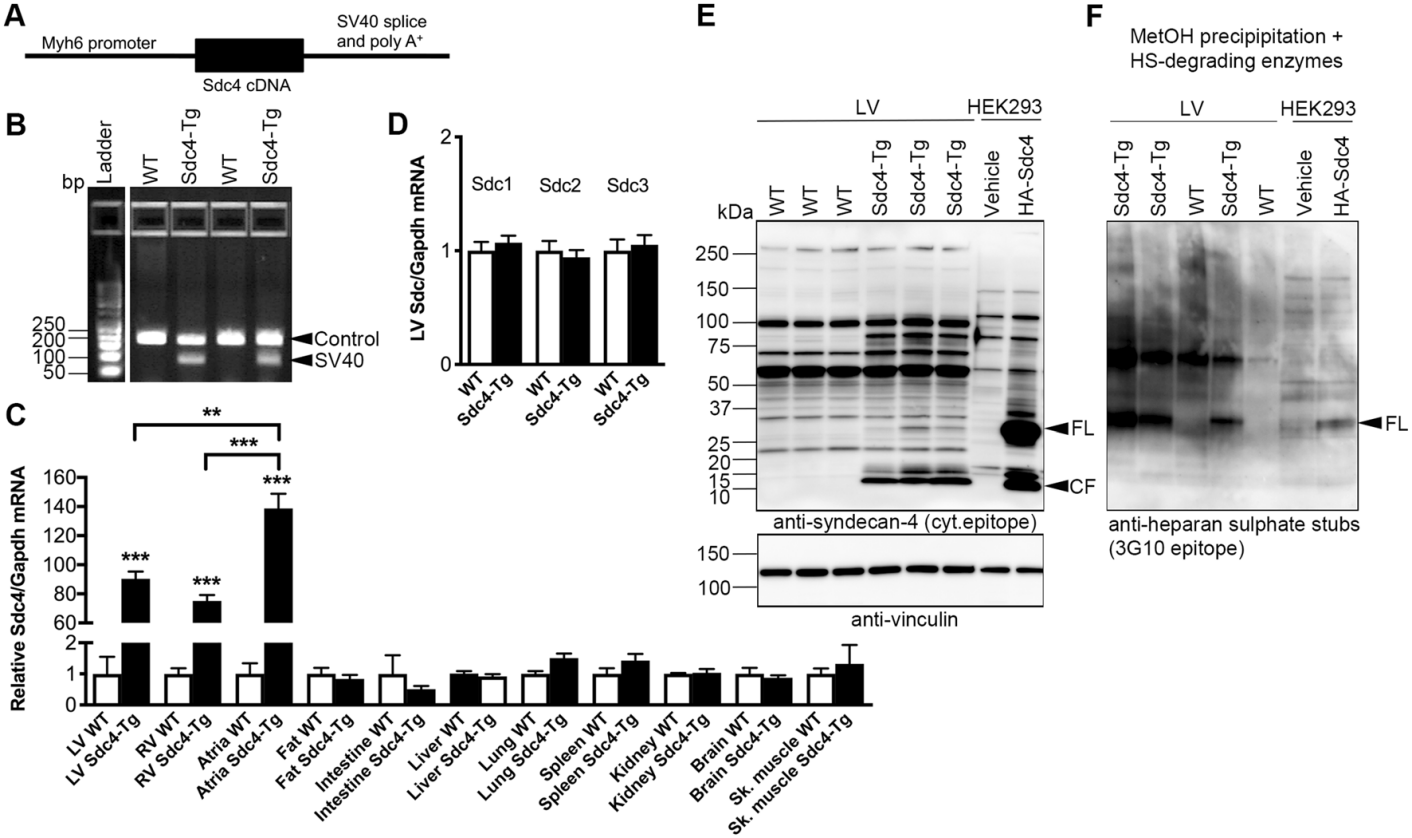
Generation of mice with cardiomyocyte-specific overexpression of syndecan-4. **A** Mice with cardiomyocyte-specific overexpression of syndecan-4 (*Sdc4-Tg*) were generated by inserting mouse *Sdc4* cDNA upstream of the mouse *Myh6* promoter, followed by the simian virus (SV40) splice acceptor site and polyadenylation signal sequence (schematic shown). A C57BL/6J mouse line with stable, germline transmission of the *Sdc4-Tg* allele was established. See Table 1 for baseline characteristics. **B** Representative genotyping gel of DNA from mice at weaning age, showing a 110 bp SV40 fragment used to identify *Sdc4-Tg* mice. A control fragment was amplified in all mice. **C** Relative mRNA expression of *Sdc4* in the left ventricle (LV), right ventricle (RV), atria, fat, intestine, lung, liver, kidney, spleen, brain and skeletal muscle (*gastrocnemius*) of *Sdc4-Tg* and WT mice (n = 3), normalized to *Gapdh*. **D** Relative mRNA expression of *Sdc1-3* in the LV, normalized to *Gapdh*. Data are mean ± SEM. Statistical differences (**C**, **D**) were tested using t-test vs. respective control tissue; **p < 0.01, ***p < 0.001. **E**, **F** Representative immunoblots of syndecan-4 in LV protein lysates (E; vinculin was used for loading control), and methanol-precipitated and heparan sulphate (HS)-digested lysates (F) from *Sdc4-Tg* and WT mice (n = 3–5), showing increased levels of syndecan-4 full-length (FL) and cellular fragment (CF) in hearts of *Sdc4-Tg* mice, the latter in line with increased syndecan-4 shedding. See Fig. S1 for syndecan-4 antibody validation. Overexpression of hemagglutinin (HA)-tagged syndecan-4 in HEK293 cells was used as positive control

To generate mice with cardiomyocyte-specific syndecan-4 overexpression, we inserted mouse *Sdc4* cDNA (NP_035651) under control of the mouse *Myh6* promoter [encoding α-myosin heavy chain (α-MHC)], followed by the simian virus (SV) 40 splice acceptor site and polyadenylation signal sequence (Fig. 1A). A 110 bp SV40 fragment was used to genotype the *Sdc4-Tg* mice (Fig. 1B). A C57BL/6J mouse line with stable, germline transmission of the *Sdc4-Tg* allele was established. *Sdc4-Tg* male and female mice had similar appearance, behaviour and fertility compared to wild type (WT) littermates. Overexpression of *Sdc4* mRNA in hearts of *Sdc4-Tg* mice was confirmed, i.e., 90-fold higher in the LV, 75-fold higher in the right ventricle (RV), and 139-fold higher in atria vs. WT (Fig. 1C). As expected from *Myh6*-driven overexpression, which is cardiomyocyte-specific, we found no difference in *Sdc4* mRNA levels in other tissues investigated, i.e., fat, intestine, liver, lung, spleen, kidney, brain or skeletal muscle. We did not observe alterations in expression of the three other syndecans in the heart (*Sdc1-3*; Fig. 1D).

While the core syndecan-4 protein is 24 kDa, syndecan-4 substituted with heparan sulphate GAGs appears in gels as bands of different sizes, in addition to variants too big to enter the gel. We have previously developed a custom made syndecan-4 antibody detecting a cytoplasmatic epitope (antibody validation in previous reports [13, 21, 23] and Fig. S1) which was used here. Immunoblotting of heart lysates showed increased levels of syndecan-4-specific protein bands including full-length (FL) syndecan-4 protein in *Sdc4-Tg* hearts vs. WT (Fig. 1E). Increased FL syndecan-4 protein was confirmed using methanol precipitation of heart lysates prior to treatment with heparan sulphate (HS)-degrading enzymes, and immunoblotting using an antibody recognizing the HS stubs [21, 23, 30] (Fig. 1F). Of note, although it is known to be expressed, syndecan-4 protein in WT hearts appeared nearly non-detectable when immunoblotted side-by-side with *Sdc4-Tg* hearts due to relatively high syndecan-4 levels in *Sdc4-Tg*.

When the extracellular domain of syndecan-4 is shed from the cell surface, the shed ectodomain (SE) is separated from the cellular fragment (CF), consisting of the transmembrane and cytoplasmic parts. The custom made syndecan-4 antibody with the cytoplasmatic epitope also detects the 10–15 kDa CF which was used to estimate syndecan-4 shedding levels (Fig. S1). *Sdc4* overexpression resulted in constitutive shedding, evident from the increased levels of the CF in *Sdc4-Tg* hearts (Fig. 1E). Thus, syndecan-4 mRNA, FL protein and shed levels were increased in hearts of *Sdc4-Tg* mice, without alterations in expression of the other three syndecan family members, and without altered syndecan-4 expression in other tissues investigated.

### *Sdc4-Tg* mice show no overt cardiac phenotype at baseline or during ageing

The cardiac phenotype of *Sdc4-Tg* mice was examined at 6–8, 30 and 60 weeks of age. We found no differences in LV weight (LVW) or lung weight (LW), or echocardiographic cardiac dimensions or function compared to WT littermates, in line with no overt cardiac phenotype of *Sdc4-Tg* at baseline or during ageing (Table 1). To investigate the molecular profiles of these hearts, expression of signature molecules of heart failure (*Nppa* and *Nppb* encoding atrial and brain natriuretic peptides (ANP and BNP), respectively), NFAT activation (*Rcan1-4* encoding the Regulator of calcineurin (RCAN) 1–4, a gene whose transcription is under the direct control of calcineurin-NFAT [31, 32]), and fibrosis, i.e., structural collagens I and III (encoded by *Col1a2* and *Col3a1*, respectively), was measured. *Sdc4-Tg* mice showed 2.6-fold increased *Nppa* and 2.0-fold increased *Nppb* expression at baseline, and 1.6-fold increased *Nppb* during aging (Fig. S2A, B, respectively), perhaps indicating a low level, pro-hypertrophic state. We found no differences in expression of *Rcan1-4* (Fig. S2C), or collagens (Fig. S2D, E).

**Table 1:** Characteristics of *Sdc4-Tg* mice at baseline and during ageing

	WT 6-8w	*Sdc4-Tg* 6-8w	WT 30w	*Sdc4-Tg* 30w	WT 60w	*Sdc4-Tg* 60w
Sex	M	M	M	M	M	M
N	13	16	10	4	10	5
BW (g)	23.35 ± 0.53	23.53 ± 0.47	32.84 ± 0.63	32.70 ± 1.55	39.77 ± 2.13	36.56 ± 1.68
LVW/BW (mg/g)	3.38 ± 0.06	3.30 ± 0.05	3.15 ± 0.08	3.19 ± 0.20	3.02 ± 0.15	2.94 ± 0.11
LW/BW (mg/g)	6.14 ± 0.46	5.82 ± 0.13	5.00 ± 0.13	5.56 ± 0.56	4.72 ± 0.22	4.80 ± 0.12
N	8	10	7	7	5	5
LAD (mm)	1.81 ± 0.06	1.80 ± 0.05	1.94 ± 0.08	1.88 ± 0.06	1.94 ± 0.03	2.03 ± 0.02
LVPWd (mm)	0.70 ± 0.02	0.70 ± 0.02	0.73 ± 0.01	0.70 ± 0.02	0.76 ± 0.03	0.74 ± 0.01
IVSd (mm)	0.72 ± 0.02	0.70 ± 0.02	0.75 ± 0.01	0.71 ± 0.01	0.76 ± 0.02	0.76 ± 0.01
LVIDd (mm)	4.10 ± 0.11	4.15 ± 0.06	4.36 ± 0.10	4.41 ± 0.15	4.54 ± 0.08	4.50 ± 0.14
FS (%)	21.66 ± 2.25	21.22 ± 1.08	22.61 ± 1.05	25.62 ± 1.12	22.59 ± 1.69	19.72 ± 1.51
Calc. LV mass (mg)	108.60 ± 4.72	108.60 ± 3.78	127.10 ± 5.18	122.40 ± 8.83	140.80 ± 6.51	136.10 ± 7.44

M-mode echocardiography data and post-mortem organ weights (mean ± SEM) of *Sdc4-Tg* and littermate wild-type (WT) male mice at 6–8, 30 and 60 weeks of age

Unpaired t-test *Sdc4-Tg* vs. WT at 6-8w, 30w and 60w, respectively

*BW* body weight, *LVW* left ventricular weight, *LW* lung weight, *LAD* left atrial diameter, *IVSd* interventricular septum thickness in diastole, *LVPWd* left ventricular posterior wall thickness in diastole, *LVIDd* left ventricular internal diameter in diastole, *FS* fractional shortening

### *Sdc4-Tg* mice show exacerbated cardiac remodelling with faster heart failure progression upon pressure overload

**Fig. 2:**
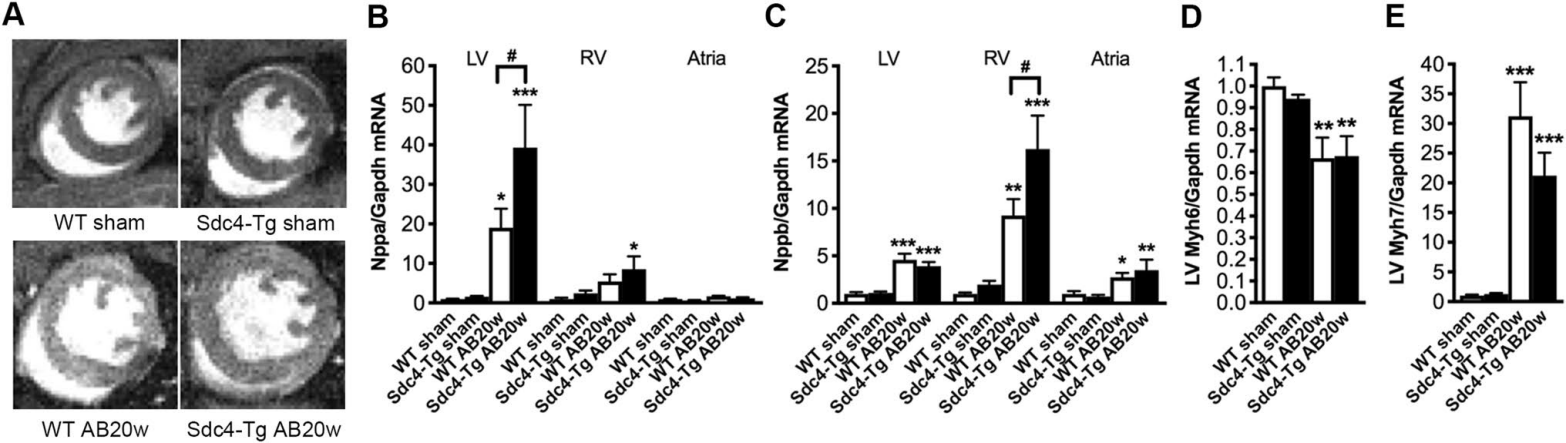
*Sdc4-Tg* mice show exacerbated cardiac remodelling upon pressure overload. **A** Representative magnetic resonance imaging (MRI), mid-ventricular, short-axis view, of *Sdc4-Tg* and wild-type (WT) mice subjected to aortic banding (AB) or sham operation for 20 weeks (20w). See Tables 2 and SI for characteristics post-AB or sham at 2, 6, 12 and 20w. Relative mRNA levels of signature molecules of heart failure, *Nppa* and *Nppb* [**B**, **C**; encoding atrial and brain natriuretic peptides (ANP and BNP, respectively)] in left ventricle (LV), right ventricle (RV) and atria of *Sdc4-Tg* and WT at 20w post-AB or sham (n = 7–9). Relative mRNA levels of markers of pathological cardiomyocyte remodelling, *Myh6* and *Myh7* [**D**, **E**; encoding α- and β-myosin heavy chain (MHC), respectively] in the LV of *Sdc4-Tg* and WT mice after 20w of AB or sham. Gene expression was normalized to *Gapdh*. Data are mean ± SEM. Statistical differences were tested using one-way ANOVA with Dunnett’s post-testing vs. respective WT sham, *p < 0.05, **p < 0.01, ***p < 0.001; or vs. WT AB20w, ^#^p < 0.05

To understand the role of syndecan-4 in cardiac hypertrophy, mice were subjected to AB or sham control operation. The cardiac phenotype was examined by standard echocardiography at 2, 6, 12 (Table SI) and 20 weeks (Table 2), with more extensive examination at termination 20 weeks post-AB, including organ weights, Doppler echocardiography and magnetic resonance imaging (MRI; Table 2 and Fig. 2A). We observed no difference in mortality between *Sdc4-Tg* to WT mice over the 20 weeks (Fig. S3A).

**Table 2:** Characteristics of *Sdc4-Tg* mice after AB

	20 weeks			
	Sham WT	Sham *Sdc4-Tg*	AB WT	AB *Sdc4-Tg*
*Biometric data*				
N	9	9	11	14
BW (g)	32.12 ± 0.67	33.09 ± 0.93	31.58 ± 0.94	29.91 ± 0.50
HW/BW (mg/g)	4.69 ± 0.29	4.38 ± 0.22	7.30 ± 0.44**	9.41 ± 0.72***^,#^
LVW/BW (mg/g)	3.13 ± 0.32	2.86 ± 0.29	5.16 ± 0.31***	6.13 ± 0.22***^,#^
RVW/BW (mg/g)	1.26 ± 0.43	1.25 ± 0.48	0.97 ± 0.07	1.12 ± 0.13
LW/BW (mg/g)	4.79 ± 0.15	4.81 ± 0.08	7.69 ± 0.63**	8.93 ± 0.64***
*M-mode echocardiography*				
N	9	9	11	14
LAD (mm)	1.66 ± 0.03	1.70 ± 0.03	2.26 ± 0.16**	2.54 ± 0.12***
LVPWd (mm)	0.73 ± 0.02	0.74 ± 0.02	0.99 ± 0.05***	1.02 ± 0.05***
IVSd (mm)	0.73 ± 0.02	0.75 ± 0.01	1.00 ± 0.04***	0.99 ± 0.03***
LVIDd (mm)	4.11 ± 0.08	4.12 ± 0.11	4.44 ± 0.13	4.96 ± 0.17***^,#^
FS (%)	31.27 ± 2.61	29.45 ± 1.98	18.77 ± 2.11***	14.88 ± 1.95***
Calc. LV mass (mg)	112.90 ± 5.43	115.30 ± 3.91	191.60 ± 11.48***	236.30 ± 14.45***^,#^
*Doppler flow echocardiography*				
N	9	9	7–11	5–13
Heart rate (BPM)	478.20 ± 16.38	487.70 ± 17.67	481.00 ± 13.65	495.80 ± 9.63
Peak mitral velocity (m/s)	624.10 ± 31.63	609.30 ± 35.48	688.90 ± 37.58	648.20 ± 61.03
Mitral deceleration (m/s)	2418 ± 159	2606 ± 198	3936 ± 333**	4121 ± 414**
CO in LVOT (ml/min)	63.33 ± 4.54	51.00 ± 2.73	57.73 ± 6.03	40.62 ± 3.58**^,#^
Peak RVOT velocity (m/s)	602 ± 23.09	666.20 ± 15.58	698.90 ± 43.21	551.20 ± 50.31#
*Tissue Doppler echocardiography*				
N	9	8	11	14
Systolic velocity (mm/s)	26.56 ± 1.80	25.00 ± 0.93	17.85 ± 1.78***	15.06 ± 0.83***
Diastolic velocity (mm/s)	25.56 ± 1.28	23.50 ± 1.38	19.63 ± 1.91*	15.94 ± 0.94***
*MRI*				
N	3	4	4	8
Calc. LV mass (mg)	126.30 ± 12.70	129.10 ± 3.85	187.50 ± 8.13***	232.70 ± 8.24***^,#^#
LV EDV (μl)	52.92 ± 4.08	60.33 ± 3.65	102.00 ± 8.45*	121.60 ± 10.60**
LV ESV (μl)	26.64 ± 6.46	30.20 ± 1.45	52.11 ± 12.24	82.75 ± 13.91*
LV EF (%)	50.63 ± 8.44	49.77 ± 1.72	50.56 ± 6.90	35.02 ± 6.03

Biometric data, M-mode, flow Doppler and tissue Doppler echocardiography, and magnetic resonance imaging (MRI) data (mean ± SEM) of male *Sdc4-Tg* and wild-type (WT) littermate control mice 20 weeks after aortic banding (AB) or sham operation. See Table SI for animal characteristics 2, 6 and 12 weeks after AB or sham operation. Blood flow over the stenosis was measured 24 h after AB: WT 3.94 ± 0.07 m/s and *Sdc4-Tg* 3.86 ± 0.08 m/s, and was not statistically different between the groups

*BW* body weight, *LVW* left ventricular weight, *LW* lung weight, *LAD* left atrial diameter, *IVSd* interventricular septum thickness in diastole, *LVPWd* left ventricular posterior wall thickness in diastole, *LVIDd* left ventricular internal diameter in diastole, *FS* fractional shortening, *CO* cardiac output, *LVOT* left ventricular outflow tract, *RVOT* right ventricular outflow tract, *LV* left ventricle, *EDV* end-diastolic volume, *ESV* end-systolic volume, *EF* ejection fraction, *BPM* beats per minute

One-way ANOVA with Dunnett´s post-test vs. WT sham; *p < 0.05; **p < 0.01; ***p < 0.001, and vs. WT AB; #p < 0.05

At 2 or 6 weeks post-AB, both genotypes developed comparable hypertrophic remodelling, evidenced by increased thickness of the LV posterior wall (LVPWd) and the interventricular septum (IVSd) in diastole, and calculated LV mass compared to sham controls (Table SI).

Interestingly, at 12 weeks post-AB, *Sdc4-Tg* mice showed increased calculated LV mass compared to WT (Table SI). Fractional shortening (FS) was reduced at 12 weeks post-AB in the *Sdc4-Tg* mice compared to WT, suggesting contractile dysfunction.

Importantly, at 20 weeks post-AB, exacerbated hypertrophy, remodelling and dysfunction in *Sdc4-Tg* mice vs. WT was evident from cardiac phenotyping (Table 2). Firstly, *Sdc4-Tg* mice showed increased heart weight (HW; 29% increase vs. WT), LVW (19% increase vs. WT) and LW (17% increase vs. WT). The increased HW and LVW were supported by estimations of LV mass from echocardiography (24% increase vs. WT) and MRI (25% increase vs. WT) (Table 2). Echocardiography showed that while LVPWd and IVSd were increased to a similar extent in *Sdc4-Tg* and WT 20 weeks post-AB, *Sdc4-Tg* mice displayed increased LV internal diameter in diastole (LVIDd) compared to WT AB, i.e., exacerbated dilatation (Fig. 2A). Both *Sdc4-Tg* and WT mice showed impaired function 20 weeks post-AB, based on reduced FS and tissue Doppler showing reduced systolic and diastolic velocities (Table 2). These measurements tended to show exacerbated dysfunction in *Sdc4-Tg,* without reaching statistical significance. As expected from heart failure signature molecules ANP and BNP, after 20 weeks of AB, *Nppa* was increased in the LV (Fig. 2B), and *Nppb* in the LV, right ventricle (RV) and atria (Fig. 2C) of both genotypes vs. sham. Supporting exacerbated heart failure, there was increased expression of *Nppa* in the LV and RV of *Sdc4-Tg* mice compared to WT after AB (Fig. 2B), and increased expression of *Nppb* in the RV (Fig. 2C). The signature molecular change of pathological cardiomyocyte remodelling, with reduced expression of *Myh6* (encoding α- MHC) and increased expression of *Myh7* (encoding β-MHC), was present in both genotypes after AB (Fig. 2D, E, respectively). Altogether, cardiac phenotyping showed that cardiomyocyte-specific overexpression of *Sdc4 *in vivo resulted in a faster progression towards dysfunction and failure after pressure overload.

### *Sdc4-Tg* mice show increased cardiac NFAT activation

**Fig. 3:**
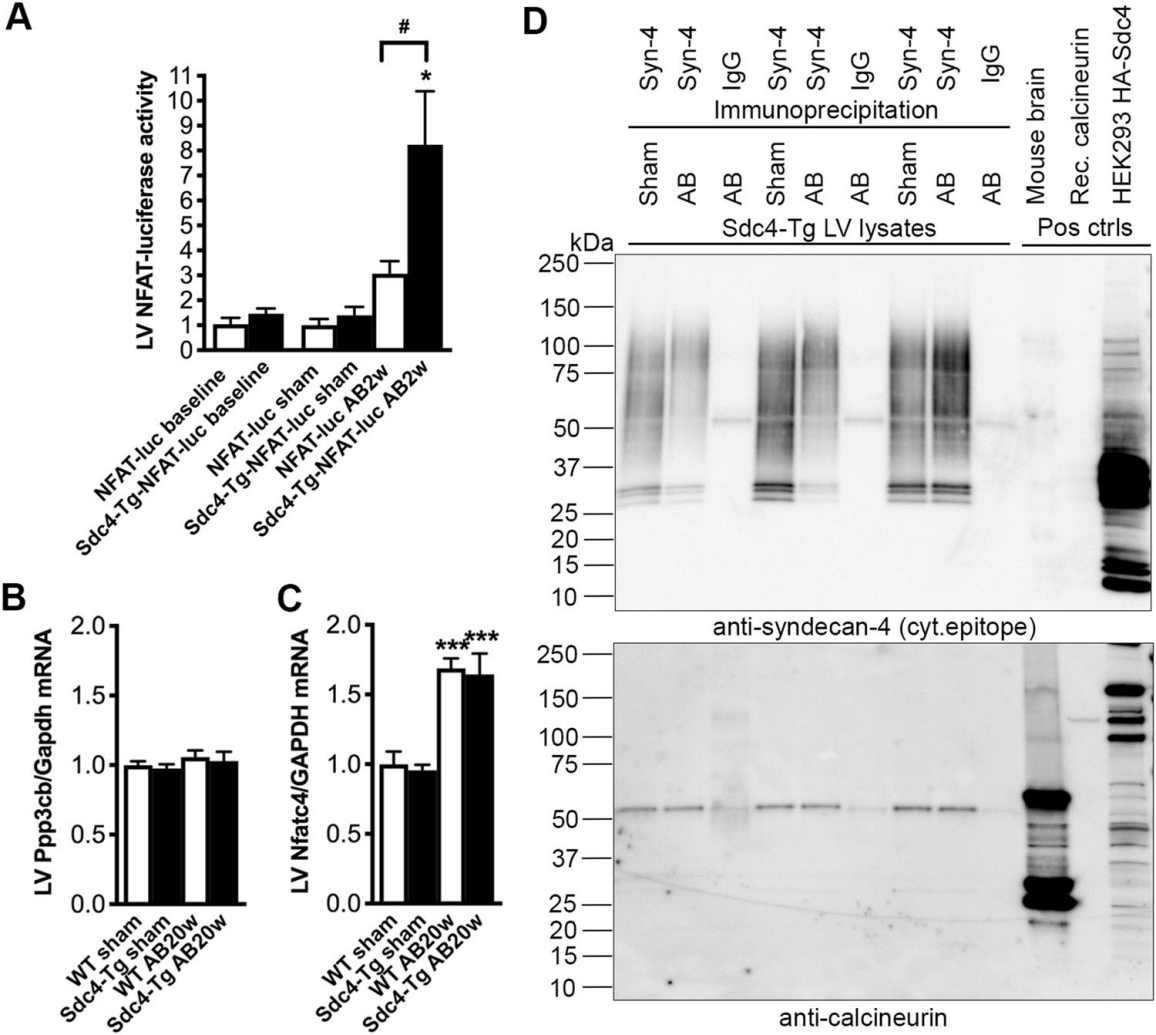
*Sdc4-Tg* mice show increased NFAT activation upon pressure overload. **A** Relative NFAT-luciferase (luc) activity in whole left ventricle (LV) of *Sdc4-Tg* -NFAT-luc and NFAT-luc mice at baseline (6–8 weeks of age, n = 8), and after two weeks of aortic banding (AB) or sham operation (n = 6–15). See Table SII for mouse characteristics. Relative mRNA levels of calcineurin (encoded by *Ppp3cb*; **B**) and nuclear factor of activated T-cells c4 (NFATc4, encoded by *Nfatc4*; **C**) in the LV of *Sdc4-Tg* and WT mice after 20 weeks of AB. Gene expression was normalized to *Gapdh*. Data are mean ± SEM. Statistical differences were tested using t-test (baseline), or one-way ANOVA with Dunnett´s post-testing vs. NFAT-luc sham, *p < 0.05; and vs. NFAT-luc AB2w, ^#^p < 0.05 (A), or using one-way ANOVA with Dunnett´s post-testing vs. respective WT sham, ***p < 0.001; and vs. WT AB20w. **D** Immunoprecipitation of full-length (FL) syndecan-4 from LVs of *Sdc4-Tg* mice, using an antibody detecting an extracellular epitope (see Fig. S1 for syndecan-4 antibody validation), or rat IgG as control. Representative immunoblots of syndecan-4 and calcineurin (n = 3). As positive controls for the calcineurin antibody, mouse brain lysate and recombinant calcineurin (130 kDa) was used. As positive control for the syndecan-4 antibody, overexpression of hemagglutinin (HA)-tagged syndecan-4 in HEK293 cells was used as positive control

To examine whether cardiomyocyte-specific overexpression of *Sdc4* affected NFAT activation in vivo, *Sdc4-Tg* mice were crossed with FVB/N NFAT-luciferase (NFAT-luc) reporter mice. NFAT activity was assessed in LV samples after 2 weeks of pressure overload of *Sdc4-Tg-NFAT-luc* and *NFAT-luc* littermates. At baseline, we found no difference in NFAT activation between *Sdc4-Tg-NFAT-luc* and *NFAT-luc* mice (Fig. 3A). Upon AB, we observed no difference in mortality in *Sdc4-Tg-NFAT-luc* mice vs. *NFAT-luc* control mice (Fig. S3B). *Sdc4-Tg-NFAT-luc* mice showed increased LV and LW weights upon pressure overload compared to *NFAT-luc* controls (Table SII), suggesting exacerbated congestive heart failure after AB in mice with *Sdc4* overexpression.

Importantly, NFAT activation was increased 8.23-fold in *Sdc4-Tg-NFAT-luc* mice after AB compared to controls; significantly more than in *NFAT-luc* after AB. Thus, mice with cardiomyocyte-specific overexpression of *Sdc4* show increased calcineurin-NFAT activation after AB.

We found no differences in mRNA expression of calcineurin (encoded by *Ppp3cb*) or NFATc4 (encoded by *Nfatc4*) between *Sdc4-Tg* vs. WT mice after AB (Fig. 3B, C, respectively). Immunoprecipitation of FL syndecan-4 in LVs from *Sdc4-Tg* mice was performed using an antibody detecting an extracellular epitope on syndecan-4 (see Fig. S1 for antibody validation), showing that calcineurin complexed with syndecan-4 to a similar degree in sham- and AB-operated mice (Fig. 3D). Thus, it was likely not increased levels of calcineurin-NFAT or calcineurin-syndecan-4 complexing that was the underlying cause of increased NFAT activation in *Sdc4-Tg* hearts after AB.

### Cardiomyocytes from adult ***Sdc4-Tg*** mice show increased diastolic Ca^2+^ levels

**Fig. 4:**
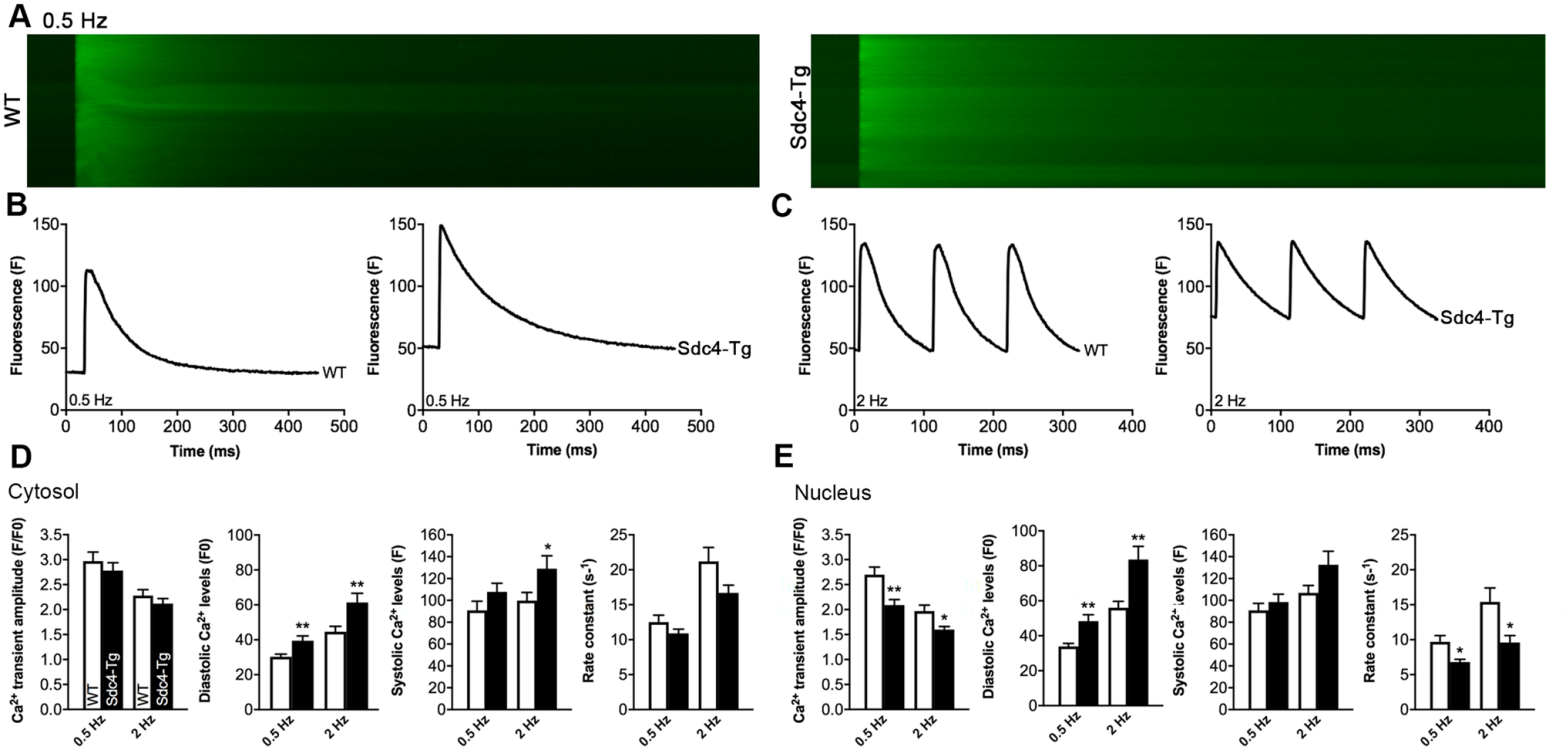
Cardiomyocytes from adult *Sdc4-Tg* mice show increased diastolic Ca^2+^ levels. **A** Representative fluorescence confocal microscopy images of cardiomyocytes isolated from adult *Sdc4-Tg* and wild-type (WT) mice, loaded with the fluorescence-labelled Ca^2+^ indicator Fluo 4-AM. Representative tracings of cells paced at 0.5 Hz (baseline condition; **B**) and 2 Hz (stressed condition; **C**). **D, E** Ca^2+^ transient characteristics of n = 16–19 cells from n = 3 WT and *Sdc4-Tg* mice, paced at 0.5 Hz and 2 Hz. Data are mean ± SEM. Statistical differences were tested using an unpaired t-test vs. respective WT control, *p < 0.05, **p < 0.01

To address whether the increased calcineurin-NFAT activation in *Sdc4-Tg* hearts was associated with altered cardiomyocyte Ca^2+^ transients, cytosolic and nuclear Ca^2+^ transients were measured in cardiomyocytes from adult *Sdc4-Tg* and WT mice, paced at 0.5 Hz and 2 Hz (Fig. 4). In cytosol (Fig. 4A–D), the diastolic Ca^2+^ levels (F0) were higher in *Sdc4-Tg* vs. WT at both 0.5 Hz and 2 Hz. Systolic Ca^2+^ levels (F) were not different at 0.5 Hz, but were higher in *Sdc4-Tg* compared to WT at 2 Hz. We found no differences in cytosolic Ca^2+^ transient amplitude (F/F0) or Ca^2+^ extrusion rate (tau) at 0.5 or 2 Hz. In the nucleus (Fig. 4A–C and E), the diastolic Ca^2+^ levels (F0) were higher in *Sdc4-Tg* vs. WT at both 0.5 Hz and 2 Hz. Systolic Ca^2+^ levels (F) were not different at 0.5 Hz, and showed a tendency towards higher Ca^2+^ levels in *Sdc4-Tg* at 2 Hz (p = 0.08). The nuclear Ca^2+^ transient amplitude (F/F0) was reduced in *Sdc4-Tg* at both 0.5 Hz and 2 Hz, with a slower Ca^2+^ extrusion rate (tau) at 0.5 Hz and 2 Hz. These experiments suggest that in cardiomyocytes from *Sdc4-Tg* mice, diastolic Ca^2+^ fluxes were altered, which likely influences calcineurin-NFAT signalling. Since transient receptor potential channels (TRPCs) 1, 3 and 6 regulate Ca^2+^-dependent calcineurin-NFAT activation in cardiomyocytes [33], we measured *Trpc 1*, *3* and *6* expression in *Sdc4-Tg* hearts. However, we found no differences in their expression levels vs. WT hearts (Fig. S4A–C, respectively).

### *Sdc4-Tg* mice did not show differences in cardiac fibrosis or immune cell infiltration upon pressure overload

To test whether overexpression of *Sdc4* in cardiomyocytes in vivo affected cardiac fibrosis, expression of *Col1a2*, *Col3a1*, the collagen cross-linking enzyme lysyl oxidase (encoded by *Lox*), connective tissue growth factor (encoded by *Ctgf*), and periostin (encoded by *Postn*) was assessed in LV, RV and atria of *Sdc4-Tg* and WT mice 20 weeks post-AB (Fig. S5A–E, respectively). Altogether, the comparable increase in these genes post-AB vs. sham suggested no difference in fibrosis in *Sdc4-Tg* hearts post-AB.

Expression of the myofibroblast signature gene *Acta2* (encoding α-smooth muscle actin) was increased to a similar extent in *Sdc4-Tg* and WT mice post-AB vs. sham (Fig. S5F), indicating no differences in myofibroblast differentiation in hearts with increased cardiomyocyte expression of syndecan-4.

To test whether overexpression of *Sdc4* in cardiomyocytes in vivo affected T-cell infiltration, expression of *Cd3* (cluster of differentiation 3), a transmembrane receptor expressed on T-cells, was assessed as in LV, RV and atria of *Sdc4-Tg* and WT mice 20 weeks post-AB. *Cd3* was increased to a comparable extent in *Sdc4-Tg* and WT hearts post-AB vs. sham (Fig. S6), suggesting similar extent of T-cell infiltration. These results indicated that overexpression of *Sdc4* in cardiomyocytes in vivo did not affect cardiac T-cell infiltration after pressure overload.

### Viral *Sdc4* overexpression in cultured cardiomyocytes results in increased calcineurin-dependent hypertrophic growth and NFAT activation

**Fig. 5:**
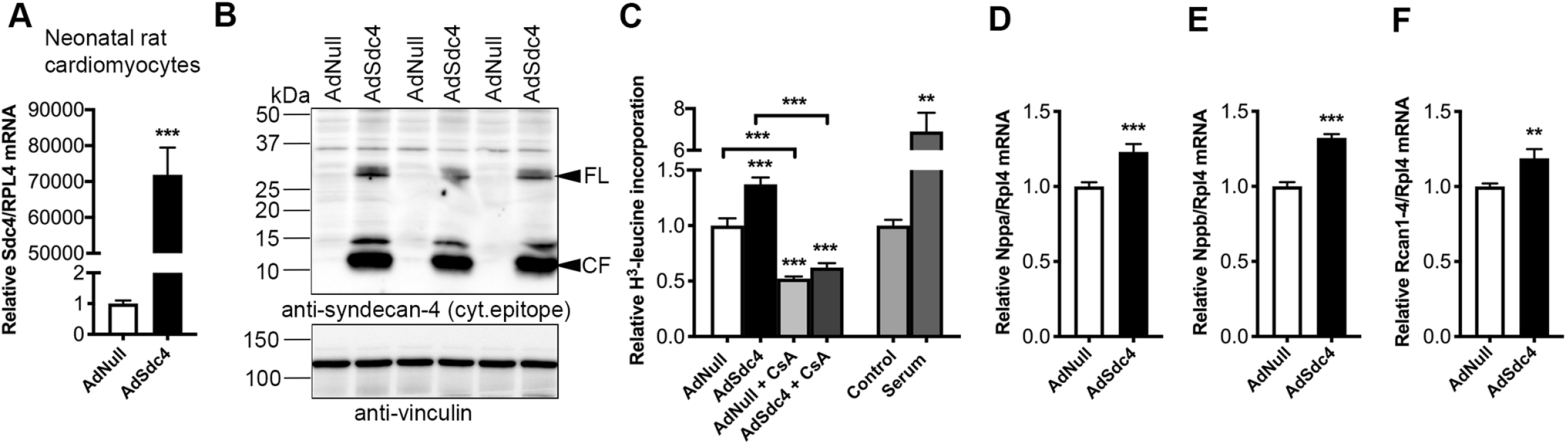
Cultured neonatal cardiomyocytes overexpressing *Sdc4* show increased calcineurin-dependent hypertrophic growth and NFAT activation. **A** Relative mRNA level of *Sdc4* in neonatal cardiomyocytes (NCM) from rats transduced with an adenovirus encoding *Sdc4* (AdSdc4) or control (AdNull), n = 11–12. **B** Representative immunoblot (n = 3) showing increased full-length (FL) syndecan-4 protein and shedding, i.e., increased levels of the cellular fragment (CF) remaining in cells after shedding of the ectodomain, in NCM protein lysates. Vinculin was used for loading control. **C** Radioactive H^3^-leucine incorporation, used to estimate protein synthesis and hypertrophic growth in vitro, in NCM transduced with AdSDC4 or AdNull (n = 8), or co-treated with Cyclosporine A (CsA, n = 5–6), an inhibitor of calcineurin. Serum-treated cells served as positive control of growth (n = 3). Relative expression of signature molecules of heart failure, *Nppa* and *Nppb* [**D**, **E**; encoding atrial and brain natriuretic peptides (ANP and BNP, respectively)], and the nuclear factor of activated T-cells (NFAT)-responsive gene *Rcan1-4* (**F**) in NCM transduced with AdSdc4 or AdNull, n = 11–12. Data are mean ± SEM. Gene expression was normalized to *Rpl4* (**A** and **C**–**E**). Statistical differences were tested using one-way ANOVA with Tukey´s multiple comparison test (C), or an unpaired t-test vs. AdNull (**A** and **D**–**F**), **p < 0.01, ***p < 0.001

To test whether overexpression of syndecan-4 induced a direct hypertrophic response in cardiomyocytes, primary cultures were prepared from neonatal rats. Neonatal cardiomyocytes (NCM) were transduced with an adenovirus encoding *Sdc4* (AdSdc4) in vitro, resulting in increased *Sdc4* mRNA (Fig. 5A) and protein (Fig. 5B) levels, both syndecan-4 FL and shedding (i.e., increased levels of syndecan-4 CF). Importantly, compared to NCM transduced with control virus (AdNull), NCM transduced with AdSdc4 showed increased protein synthesis measured by radioactive H^3^-leucine incorporation, in line with increased hypertrophic growth in vitro (Fig. 5C). The increased protein synthesis resulting from *Sdc4* overexpression was attenuated when cells were co-treated with the calcineurin inhibitor Cyclosporine A (CsA), suggesting that the *Sdc4*-induced protein synthesis in NCM was calcineurin-dependent (Fig. 5C). Increased mRNA expression of *Nppa* (Fig. 5D) and *Nppb* (Fig. 5E) supported the finding of increased hypertrophic growth, and increased *Rcan1-4* levels (Fig. 5F) indicated increased cardiomyocyte calcineurin-NFAT signalling.

Finally, we tested whether shed syndecan-4 ectodomains (SE) affected cardiomyocyte growth and NFAT signalling in vitro. Syndecan-4 SE was produced in human embryonic kidney (HEK) 293 cells transfected with a plasmid encoding HA-tagged *Sdc4,* which results in constitutive shedding evidenced by increased levels of CF in cell lysates (Fig. S1A) and SE in the medium (Fig. S1B). Cultured NCM were treated with conditioned medium from the HEK293 cells. Comparing NCM treated with shed syndecan-4 to NCM treated with vehicle control conditioned media, we found no differences in expression of *Nppa*, *Nppb* or *Rcan1-4* (Fig. S7A–C, respectively). Thus, the observed effects of *Sdc4* overexpression on cardiomyocyte hypertrophy and NFAT signalling were likely mediated through the FL syndecan-4 protein, and not shed syndecan-4 ectodomains.

## Discussion

To examine the role of syndecan-4 in cardiomyocyte hypertrophy and activation of the Ca^2+^-dependent calcineurin-NFAT pathway, we developed a mouse line with cardiomyocyte-specific *Sdc4* overexpression. *Sdc4-Tg* mice showed no overt cardiac phenotype without stress. Importantly, *Sdc4-Tg* mice showed exacerbated cardiac remodelling upon pressure overload. At 2–6 weeks post-AB, *Sdc4-Tg* and WT mice developed comparable hypertrophic remodelling, however, at 12 weeks, *Sdc4-Tg* showed worse contractile dysfunction. At 20 weeks post-AB, exacerbated hypertrophy, remodelling and dysfunction in *Sdc4-Tg* mice was evident from post-mortem organ weights, echocardiography and MRI. Using NFAT-luciferase reporter mice, we found that NFAT activation was increased in *Sdc4-Tg* hearts after AB. Immunoprecipitation showed that calcineurin bound to syndecan-4 to a similar degree in sham- and AB-operated *Sdc4-Tg* mice. Isolated cardiomyocytes from adult *Sdc4-Tg* mice showed increased diastolic Ca^2+^ levels, suggesting that syndecan-4 regulates Ca^2+^ levels, and that elevated Ca^2+^ levels activate the syndecan-4-calcineurin complex, resulting in NFAT activation and hypertrophic growth. Primary cardiomyocyte cultures from neonatal rats showed increased calcineurin-NFAT-dependent hypertrophic growth upon viral *Sdc4* overexpression. Taken together, cardiomyocyte-specific overexpression of *Sdc4* results in hypertrophic remodelling with activation of Ca^2+^-dependent calcineurin-NFAT signalling, resulting in a faster progression towards dysfunction after pressure overload.

Syndecan-4 localizes to the costamere and Z-disc of cardiomyocytes [28], areas linking the cytoskeleton to the ECM, that are believed to be important for sensing mechanical stress. Syndecan-4 mRNA and protein levels are elevated in hypertrophic and failing hearts of patients with AS and mice after AB [13, 19–23]. We have shown that in AS patients, shed syndecan-4 is detected at higher levels in the coronary sinus than in peripheral blood [23], suggesting it is shed from the hypertrophic human heart. Serum syndecan-4 levels are elevated in patients with heart failure [34, 35], increase in proportion with LV mass, and correlate with LV geometrical parameters [35], suggesting it could have value as a blood biomarker. Mimicking the elevated syndecan-4 mRNA, protein and shedding in the myocardium of AS patients, syndecan-4 mRNA, protein and shedding was elevated in *Sdc4-Tg* hearts. Syndecans are involved in a variety of functions [5, 10, 12], and our understanding of syndecan-4 in cardiac remodelling has mainly come from studies of constitutive *Sdc4-KO* mice [13, 14, 19–22]. As syndecan-4 is expressed at comparable levels in cardiac myocytes and fibroblasts [19], its specific role in cardiomyocytes has remained incompletely understood. To tease this out, we developed *Sdc4-Tg* mice with cardiomyocyte-specific syndecan-4 overexpression. Bearing in mind that these mice have relatively high cardiomyocyte syndecan-4 expression, mice with cardiomyocyte-specific KO should be developed in the future.

The *Sdc4-Tg* mice gave us the novel insight that syndecan-4 directs Ca^2+^-dependent calcineurin-NFAT activation and hypertrophic remodelling after pressure overload in cardiomyocytes in vivo. NFAT activation was increased in *Sdc4-Tg* hearts, which is in line with reduced NFAT activation and hypertrophic remodelling in hearts of *Sdc4-KO* mice [13]. In cultured cardiomyocytes, we found that viral overexpression of syndecan-4 resulted in NFAT activation and hypertrophic growth. This is in line with reduced NFAT activation and hypertrophic growth in cardiomyocytes from *Sdc4-KO* mice and increased NFAT activation in WT cardiomyocytes treated with a syndecan-4 peptide [13], however it contrasts a study of cultured cardiomyocytes with reduced syndecan-4 expression, where NFAT activation was increased [14]. Altogether, we believe that syndecan-4 is an activator of cardiomyocyte NFAT signalling.

NFAT transcription factors are activated specifically by dephosphorylation by the Ca^2+^-activated phosphatase calcineurin [26]. We have identified that calcineurin binds directly to the cytoplasmic domain of syndecan-4 though its autoinhibitory domain, a domain regulating calcineurin activity, and that this binding is elevated in WT mice after AB [13]. We here confirmed that calcineurin binds to syndecan-4 in cardiomyocytes. That level of binding was comparable in sham- and AB-treated mice could be due to the relatively high syndecan-4 levels in *Sdc4-Tg* mice, however, this also implies that the binding itself is not sufficient for calcineurin activation by syndecan-4. We have previously identified that the Ca^2+^-binding co-activator calmodulin (CaM) is localized to the syndecan-4-calcineurin complex in mouse hearts [13], suggesting that the syndecan-4-mediated calcineurin activation is Ca^2+^-dependent. We here found increased diastolic levels of Ca^2+^ in cardiomyocytes from *Sdc4-Tg* hearts. Ca^2+^ binds to calmodulin, resulting in calmodulin modifying its interactions with target proteins, e.g., calcineurin. Calcineurin avoids activation by cardiac cycle beat-to-beat Ca^2+^ oscillations, but is activated by cytosolic levels elevated beyond a threshold, typically found in heart failure [36]. Thus, we believe that elevated Ca^2+^ levels found in cardiomyocytes with high syndecan-4 levels, likely through calmodulin binding, is a probable mechanism for NFAT activation, both through syndecan-4-bound and *non*-syndecan-4-bound calcineurin.

Although care should be taken when interpreting these results, the increased Ca^2+^ levels in *Sdc4-Tg* cardiomyocytes could be a result of direct effects of syndecan-4 on cardiomyocyte Ca^2+^ channels. Syndecan-4 has been linked to regulation of cellular Ca^2+^ levels through transient receptor potential channels (TRPCs) [37]. Although we found no differences in expression of *Trpc 1, 3* and *6* in *Sdc4-Tg* hearts, more detailed studies are needed to elucidate whether syndecan-4 regulates Ca^2+^ fluxes through these or other ion channels in cardiomyocytes, and thereby, cardiomyocyte calcineurin-NFAT activation.

Since we and others have established that syndecan-4 is important for fibroblast function, formation of focal adhesions, fibrosis and immune cell infiltration [7–9, 21, 29], we could not rule out from studies of full-body *Sdc4-KO* mice that cardiomyocyte effects were not secondary to fibroblast-mediated changes or inflammation. Syndecan-4 is located to focal adhesions in fibroblasts, linking the cytoskeleton to the ECM [7, 8, 29, 38], and regulates cardiac myofibroblast transdifferentiation and production of ECM constituents, including collagens, and collagen cross-linking [19, 20]. Syndecan-4 also regulates infiltration of immune cells to the heart [21, 23, 39]. Our results from cardiomyocyte-specific syndecan-4 overexpression suggest that syndecan-4-mediated fibrosis is a direct effect of increased levels in fibroblasts, and that immune cell infiltration likely is not directed by increased levels in cardiomyocytes.

In conclusion, we here developed mice with cardiomyocyte-specific overexpression of the transmembrane heparan sulphate proteoglycan syndecan-4, and identified that syndecan-4 is important for activation of Ca^2+^-dependent calcineurin-NFAT signalling, hypertrophic remodelling and dysfunction in cardiomyocytes in response to pressure overload. These findings extend our understanding of the important role that syndecan-4 plays in the failing heart.

## Methods

### Ethics

Animal protocols were reviewed and approved by the Norwegian National Animal Research Committee (protocol IDs 4216, 8617 and IV 1-17U) and conformed to the NIH Guide for the Care and Use of Laboratory Animals (NIH publication no. 85-23, revised 2011). Reporting of procedures and results was in accordance with the Animal Research: Reporting of In Vivo Experiments (ARRIVE) guidelines [40].

### Mouse lines

A DNA fragment encoding the entire open reading frame of mouse *Sdc4* cDNA (NP_035651) was inserted into a pBluescript plasmid vector downstream of the mouse *Myh6* promoter, and upstream of the SV40 splice acceptor site and polyadenylation signal sequence. Correct *Sdc4* insert was confirmed by sequencing, and expression in HEK293 cells was confirmed after transferal to a pCMV-SPORT6.1 backbone (Addgene, MA), with transfection using Lipofectamine2000 (Invitrogen, UK). Embryo injection of the *Myh6-Sdc4* construct was performed at the Karolinska Institutet Mouse Models (KIMM) facility, Karolinska Institutet, Stockholm, Sweden, yielding two transgenic mice confirmed by genotyping. Upon breeding with C57BL/6JBomTac (Taconic, Skensved, Denmark), a C57BL/6J mouse line with stable, germline transmission of the *Sdc4-Tg* allele was established. Heterozygous *Sdc4-Tg* and WT littermates from F2 generation and onwards were used for experiments. Genotyping was performed at weaning age with DNA isolated from ear biopsies, amplifying a 110 bp SV40 fragment by polymerase chain reaction (PCR) to identify *Sdc4-Tg* mice (primers: F1_5′- CAGTGGTGGAATGCCTTTAATGA-3′, R1_5′- AGGAGTAGAATGTTGAGAGTCAGCAGTA-3′), and a 200 bp control fragment in the *Fabpi* gene present in all mice (primers: F2_5′-TGGACAGGACTGGACCTCTGCTTTCTTAGA– 3′, R2_5′-TAGAGCTTTGCCACATCACAGGTCATTCAG-3′). To confirm specific syndecan-4 expression in the heart, LV, RV, atria, intestine, spleen, skeletal muscle (*gastrocnemius* muscle), lung, liver, brain and kidney were harvested from male mice. *NFAT-luciferase* (*NFAT-luc*) reporter mice (FVB/N) [27] were kindly provided by Professor Jeffery D. Molkentin (Cincinnati Children’s Hospital Medical Center, Cincinnati, OH). These mice carry nine copies of NFAT-binding sites from the *IL-4* promoter upstream of the luciferase reporter gene. Homozygous *NFAT-luc* mice were crossed with heterozygous *Sdc4-Tg* mice to generate heterozygous *Sdc4-Tg-NFAT-luc* mice and *NFAT-luc* littermate controls on mixed background. Male mice were used.

### Aortic banding

Banding of the ascending aorta of 6–8 week old male *Sdc4-Tg*, WT, *Sdc4-Tg-NFAT-luc* and *NFAT-luc* mice was performed by an experienced researcher blinded to genotype, as described [21, 41, 42]. Sham-operation consisted of the same procedure without tightening of the suture. During surgery, mice were intubated and ventilated with a mixture of 98% oxygen and 2% isoflurane. Animals received post-operative analgesia by subcutaneous injection of 0.02 ml buprenorphine (0.3 mg/ml).

### Cardiac phenotyping

Echocardiography of mice breathing 1.75% isoflurane on a mask was performed using the VEVO 2100 system (VisualSonics, Toronto, Canada) by an experienced researcher blinded to genotype, as described [21, 23, 42]. *Sdc4-Tg* and WT mice were examined by echocardiography at baseline (6–8 weeks of age) and during aging (30 and 60 weeks of age). Echocardiography with Doppler flow 24 h after AB was used to include mice with a maximal flow velocity over the stenosis (Vmax) of 3.5–4.5 m/s. *Sdc4-Tg* and WT mice were followed with serial echocardiography at 2, 6, 12 and 20 weeks post-AB. LVPWd, IVSd, LVIDd and systole (LVIDs) were obtained from M-mode recordings. LV mass and FS were calculated from M-mode images: 1.05*(IVSd + LVPWd + LVIDd)^3^ −  (LVIDd^3^), and 100*((LVIDd-LVIDs)/LVIDd), respectively. Left atrial diameter (LAD) was measured in atrial diastole. Tissue Doppler echocardiography was used to obtain maximal systolic and diastolic tissue velocities, and Doppler flow echocardiography was used to examine peak mitral velocity, mitral deceleration, peak RV outlet tract (RVOT) velocity. Cardiac output (CO) in LV outlet tract (LVOT) was calculated from heart rate, LVOT velocity time integral (VTI) and LVOT diameter.

MRI was performed using a 9.4T preclinical MR system (Agilent Technologies Inc., CA) in a randomly selected subset of *Sdc4-Tg* and WT 20 weeks after sham or AB, by an experienced researcher blinded to genotype, as described [43, 44]. Mice were sedated and mask ventilated 1.0–1.5% isoflurane, and body temperature was maintained by heated air guided by continuous body temperature recoding. Apical-to-basal LV short-axis slices (7–10 slices of thickness 1.0 mm) were acquired, and LV mass calculated as the sum of LV wall thicknesses from all slices *1.05, from slices obtained both in systole and diastole. End-diastolic volume (EDV) and end-systolic volume (ESV) were calculated as the sum of lumen diameter from all slices, from slices obtained in diastole and systole, respectively. Ejection fraction (EF) was calculated by the following formula: (100*(EDV − ESV)/EDV).

Mice were sacrificed by cervical dislocation under deep anaesthesia. Tissues were rapidly excised, rinsed in 1X phosphate-buffered saline (PBS) and blotted dry, snap-frozen in liquid nitrogen and stored at -70 °C. Atria, RV and LV were rapidly dissected and snap-frozen for molecular analyses. No tissue was prepared for histology. LVW, RV weight (RVW) and LW were normalized to body weight (BW).

### Left ventricular NFAT-luciferase activity

Frozen, whole LVs from *Sdc4-Tg-NFAT-luc* and *NFAT-luc* mice were homogenized twice at 30 Hz for 100 s using 5 mm stainless steel beads and the TissueLyser II (Qiagen), according to the Luciferase Assay System protocol (Promega, WI). Samples were kept on ice, vortexed and centrifuged at 12 000×*g* for 30 s. Luminescence from was quantified on the Hidex Sense Microplate Reader (Finland).

### Ca^2+^ transients in isolated adult ventricular cardiomyocytes

Cardiomyocytes from adult male *Sdc4-Tg* and WT mice were isolated as described [45]. Mice were anaesthetized in 4% isoflurane, 65% N_2_O and 31% O_2_, and intubated and ventilated with 2% isoflurane, 66% N_2_O and 32% O_2_. The heart was excised and immediately cooled in buffer A (Hepes 25 mM, NaCl 130 mM, KCl 5.4 mM, NaH_2_PO_4_ 0.4 mM, MgCl_2_ 0.5 mM, D-glucose 22 mM, pH 7.4) at 4 °C. Cardiomyocytes were isolated by direct needle perfusion of the LV for 20–25 min with buffer A containing collagenase II (Worthington Biochemical Corporation, US). Following sedimentation of cardiomyocytes, the cells were washed twice in buffer A containing (1) 0.1% bovine serum albumin (BSA) and 0.1 mmol/L CaCl_2_, and (2) 0.1% BSA and 0.2 mmol/L CaCl_2_.

Cells were loaded with 5 uM Fluo 4-AM for 30 min, before superfused with a solution containing (in mmol/L): NaCl 140, Hepes 5, KCl 5.4, CaCl_2_ 1, MgCl_2_ 0.5, D-glucose 5.5 and NaH_2_PO_4_ 0.4. pH, adjusted to 7.4 with NaOH. All experiments were performed at room temperature. Cytosolic and nuclear Ca^2+^ transients were obtained simultaneously in field-stimulated cells at 0.5 and 2 Hz using a line scan on a confocal microscope. Cardiomyocytes were scanned every 1.5 ms by an LSM 7 Live scanning system (Zeiss, Germany), with a 512 pixel line drawn along the longitudinal axis of the cell, including the nucleus and cytosol, as previously described [46]. All settings, including gain, were kept stable between experiments. Ca^2+^ transients were analysed in ImageJ (NIH, MD), and the rate constant was calculated from *tau* values obtained using Clampfit 10.4 monoexponential fitting of the Ca^2+^ extrusion phase (Axon instruments, Union City, CA).

### HEK293 cell cultures and transfection

HEK 293 cells were cultured and transfected using Lipofectamine 2000 (Invitrogen) with human influenza hemagglutinin (HA)-tagged mouse *Sdc4* (NP_035651; HA-Sdc4) in a pcDNA3.1 plasmid (Invitrogen; custom made by Genscript Corporation, NJ), as described [13, 21, 23]. Transfection with empty pcDNA3.1 plasmid (vehicle) and non-transfected cells served as controls. HEK293 cells were harvested 24 h after transfection, and samples stored at − 70 °C. Cell protein lysates and medium were used as positive controls and for antibody validation by immunoblotting. Conditioned medium from transfected HEK293 cells was also used to treat cultured cardiomyocytes from neonatal rats to study effects of shed syndecan-4 fragments. The conditioned medium was cleared by centrifugation at 5000×*g*.

### Neonatal cardiomyocyte cultures

Ventricular cardiomyocyte cultures were prepared from hearts of neonatal (1–3 days old) Wistar rats (Taconic), as described [21, 23, 47]. Hearts were trimmed of atrial tissue and digested mechanically in a collagenase/pancreatin solution, and transferred to uncoated culture flasks with serum-containing medium for 20 min, allowing fibroblasts to attach. Unattached cells, i.e., cardiomyocytes, were transferred to 6-well dishes coated with gelatin/fibronectin at a density of 3.75 × 10^5^/ml medium. Cells were kept in a 37 °C, 5% CO_2_ humidified incubator. The purity of similar cultures has been confirmed by an 800-fold higher expression of cardiac troponin I (*TnnI*) in cardiomyocytes compared to fibroblasts [21]. Cells from three separate cell culture isolations were used.

NCM were transduced with a human adenovirus type 5 encoding mouse *Sdc4* under control of the CMV promoter (Ad-mSDC4, ADV-271493) or empty vector control (Ad-CMV-Null, #1300), both from Vector Biolabs, for 48 h, with serum deprivation during the final 24 h, as described [42]. Virus titer was 5 × 10^6^ plaque forming units (PFU)/ml medium. Cells were co-treated with the calcineurin inhibitor Cyclosporine A (CsA). Cells were harvested and mRNA and protein stored at − 70 °C.

In a separate set of experiments, following 24 h of serum deprivation, NCM were treated for 24 h with 2 ml conditioned medium (diluted 2:1 in fresh medium) from HA-Sdc4 or vehicle-expressing HEK293 cells, containing shed syndecan-4 ectodomains or control, respectively, as described [23]. A limitation to these experiments is that the glycosylation of syndecan-4 may differ between human embryonic kidney and NCM. Cardiomyocytes were harvested and mRNA stored at − 70 °C.

### Neonatal cardiomyocyte protein synthesis assay

The radioactive [^3^H] leucine incorporation protein synthesis assay was performed as described [42, 48]. NCM were virally transduced in serum-containing medium for 24 h prior to culturing in serum-deprived medium containing 1.25 µCi/ml [^3^H] leucine (American Radiolabel Chemicals, MO) for 72 h. At harvest, cardiomyocytes were washed in 95% ethanol and lysed in 0.2 M NaOH. Lysates were diluted in Pico-Fluor 40 (PerkinElmer) and [^3^H] leucine incorporation quantified as counts per minute (CPM) using the Wallac Winspectral 1414 liquid scintillation counter (PerkinElmer, MA). Samples were measured in duplicates, and serum was used as positive control.

### RNA isolation and quantitative real-time PCR

RNA was extracted from frozen LV tissue or cultured cardiomyocytes using the RNeasy Mini Kit (Qiagen). Reverse transcription of 2 μg RNA was performed using the iScript cDNA Synthesis Kit (BIO-RAD). Pre-designed TaqMan assays (Applied Biosystems, CA) were used to determine gene expression (Table SIII). Results were detected on a 7900HT Fast Real Time PCR System, and data analysed using Sequence Detection Software 2.3 (Applied Biosystems).

### Protein isolation, immunoprecipitation and immunoblotting

Protein lysates were prepared from frozen LV tissue or cultured cardiomyocytes with ice cold 1X PBS-based lysis buffer (1% Triton X-100 (Sigma, MI), 0.1% Tween-20 (Sigma), protease (Complete EDTA-free tablets) and phosphatase inhibitors (PhosSTOP; both from Roche Diagnostics, Germany). Tissue samples were homogenized twice at 30 Hz for 100 s using 5 mm stainless steel beads and the TissueLyser II, left on ice for 30 min and centrifuged at 20 000×*g* for 10 min at 4 °C.

Analysis of syndecan-4 protein after precipitation of proteoglycans and GAG digestion was performed as described [21, 23, 30]. Proteoglycans were precipitated from 100 μg LV protein lysates or 40 μg cell protein lysates in methanol overnight at − 20 °C, and the pellet was resuspended in heparitinase buffer with haparitinase I–III and chondroitinase cABC (Amsbio, UK). Samples were incubated twice for 2 h at 37 °C, with addition of second and third aliquots of enzymes.

Immunoprecipitation from LV protein lysates was performed using Dynabeads, according to protocol (14311D, Thermo Fischer Scientific), using 7 μg of a syndecan-4 antibody (BD550351, BD Biosciences) detecting an extracellular epitope (see Fig. S1) or rat IgG (sc-2026, Santa Cruz Biotechnology) as negative control. Immunoprecipitates were analysed by immunoblotting.

SDS-PAGE and blotting was performed according to the Criterion protocol (BIO-RAD), as described [13, 48]. Blots were blocked in 8% dry-milk (BIO-RAD), 5% BSA (BIO-RAD) or 1% casein (Roche Diagnostics) and incubated with antibodies diluted in 2% dry-milk/5% BSA/1% casein. Blots were developed using ECL Plus Western Blotting Detection System (GE Healthcare, UK) in the Las-4000 mini (Fujifilm, Japan), stripped (Restore Western Blot Stripping Buffer, Thermo Scientific) and reprobed. Quantification and processing were performed using Image J (NIH) and Adobe Photoshop CC 2019. Primary antibodies used are found in Table SIII. Syndecan-4 antibodies were validated in cell lysates and medium from HEK293 cells overexpressing syndecan-4 (Fig. S1). A custom made antibody recognizing the cytoplasmic epitope of syndecan-4 [13, 21, 23] was used to detect FL syndecan-4 and the transmembrane plus cytoplasmic part of syndecan-4 (10–15 kDa) remaining in cells after shedding of its ectodomain, the latter used to estimate shedding in cell or LV lysates. HRP-conjugated secondary antibodies (Southern Biotechnology, AL) were applied to all blots.

### Statistics

Data are expressed as group means ± standard error of the mean (SEM). Graphs were made and statistical differences tested in GraphPad Prism 9, with p < 0.05 was considered significant. The specific statistical tests used are described in the figure legends.

## Supplementary Information

Below is the link to the electronic supplementary material.

Supplementary file1 (DOCX 25280 KB)

## Abbreviations

AB: Aortic banding
AS: Aortic stenosis
ARRIVE: Animal research: reporting of in vivo experiments
ANP: Atrial natriuretic peptide
BW: Body weight
BSA: Bovine serum albumin
BNP: Brain natriuretic peptide
CO: Cardiac output
CF: Cellular fragment
CPM: Counts per minute
CsA: Cyclosporine A
EF: Ejection fraction
EDV: End-diastolic volume
ESV: End-systolic volume
ECM: Extracellular matrix
FS: Fractional shortening
FL: Full-length
GAG: Glycosaminoglycan
HA: Hemagglutinin
HW: Heart weight
HS: Heparan sulphate
HEK: Human embryonic kidney
IVS: Interventricular septal thickness
KO: Knock-out
LAD: Left atrial diameter
LV: Left ventricle
LVID: Left ventricular internal diameter
LVOT: Left ventricular outflow tract
LVPW: Left ventricular posterior wall thickness
LVW: Left ventricular weight
LW: Lung weight
luc: luciferase
MRI: Magnetic resonance imaging
MHC: Myosin heavy chain
NCM: Neonatal cardiomyocyte
NFAT: Nuclear factor of activated T-cell
PBS: Phosphate-buffered saline
PCR: Polymerase chain reaction
RCAN: Regulator of calcineurin
RV: Right ventricle
RVOT: Right ventricular outflow tract
SV: Simian virus
SE: Shed ectodomain
VTI: Velocity time integral
