# Supplementary material for: Cardiomyocyte-specific overexpression of syndecan-4 in mice results in activation of calcineurin-NFAT signalling and exacerbated cardiac hypertrophy
Source: Mol Biol Rep. 2022 Oct 7;49(12):11795–809. doi: 10.1007/s11033-022-07985-y (PMC9712407; doi:10.1007/s11033-022-07985-y)
Supplement: Supplementary file 1 — Supplementary file1 (DOCX 25280 KB) [file 11033_2022_7985_MOESM1_ESM.docx]

**Supplemental Material**

**Cardiomyocyte-specific overexpression of syndecan-4 in mice results in activation of calcineurin-NFAT signalling and exacerbated cardiac hypertrophy**

Ida G. Lunde^1,2,3,#^, J. Magnus Aronsen^1,4^, A. Olav Melleby^1,4^, Mari E. Strand^1,2^, Jonas Skogestad^1,4^, Bård A. Bendiksen^1,2^, M. Shakil Ahmed^5^, Ivar Sjaastad^1,2^, Håvard Attramadal^5^, Cathrine R. Carlson^1,2^, Geir Christensen^1,2^

^1^ Institute for Experimental Medical Research, Oslo University Hospital and University of Oslo, Oslo, Norway.

^2^ KG Jebsen Center for Cardiac Research, University of Oslo, Oslo, Norway.

^3^ Division of Diagnostics and Technology, Akershus University Hospital, Lørenskog, Norway.

^4^ Institute for Medical Biosciences, University of Oslo, Oslo, Norway.

^5^ Institute for Surgical Research, Oslo University Hospital and University of Oslo, Oslo, Norway.

**Supplemental Figures**

**
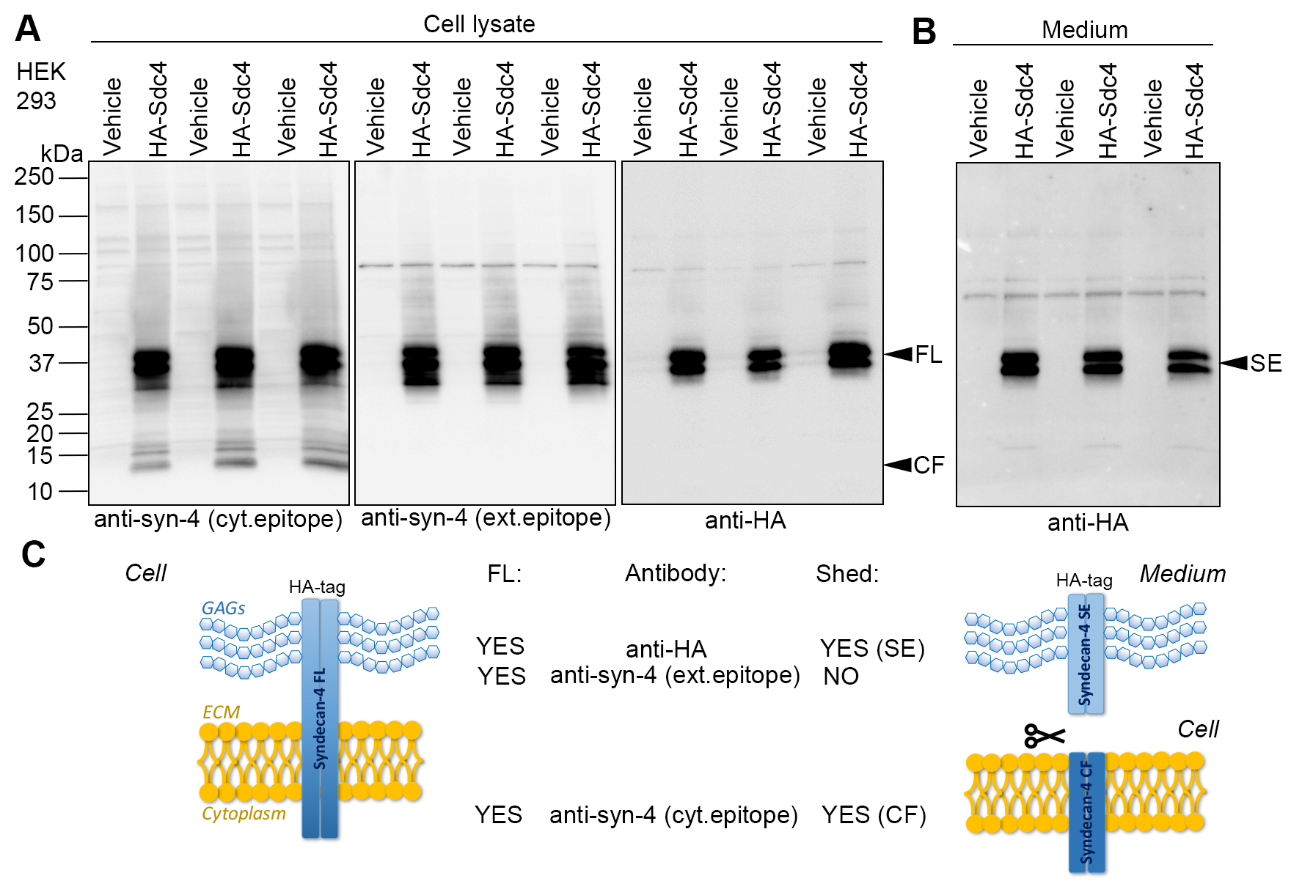
**

**Fig. S1 Validation of antibodies to detect full-length and shed syndecan-4 protein.**

Representative immunoblots of **A** cell lysate and **B** cell medium from HEK293 cells transfected with *Sdc4* tagged with influenza hemagglutinin (HA) N-terminally, or vehicle control plasmid (n=3). The syndecan-4 antibody recognizing a cytoplasmic (cyt.) epitope (Genscript, custom made [1]) has been characterized previously (for details, see Strand *et al*. [2, 3]), while the antibody detecting the extracellular (ext.) epitope was commercially available (BD550351, BD Biosciences). **C** Schematic of full-length (FL) syndecan-4 (left) and shedding (right), with indications of successful antibody detection indicated as YES or NO. FL, transmembrane syndecan-4 was detected in cellular lysates by the antibodies detecting the cyt. and the ext. epitopes, and the antibody detecting the N-terminal HA-tag. Shedding was detected in cell medium by the anti-HA antibody. Of note, the syndecan-4 antibody detecting the ext. epitope did not successfully detect the shed ectodomain (SE) in medium (data not shown). Shedding was detected in cell lysate by the antibody detecting the cyt. epitope, detecting the cellular fragment (CF) remaining in cell after release of the SE. Thus, level of CF was used to estimate endogenous syndecan-4 shedding due to lack of an antibody detecting the SE. For immunoprecipitation of FL, the syndecan-4 antibody detecting the ext. epitope was used.

**
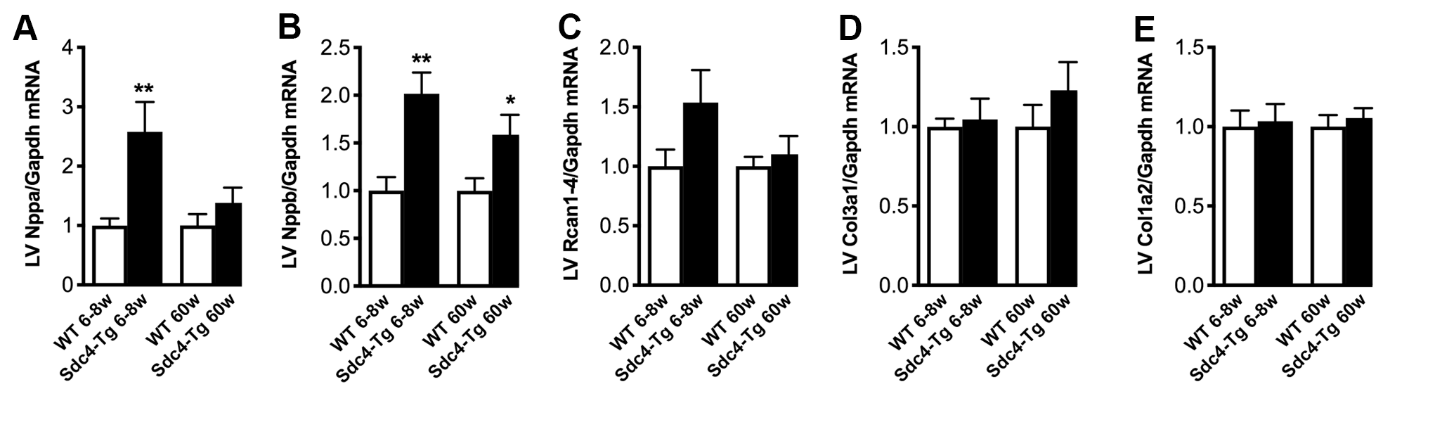
Fig. S2 Expression of signature molecules of heart failure, NFAT activation and fibrosis in left ventricles of young and aged, untreated *Sdc4-Tg* mice**

Relative mRNA expression of signature molecules of heart failure (*Nppa* and *Nppb* encoding atrial and brain natriuretic peptides; **A-B)**, NFAT activation (*Rcan1-4*; **C**), and fibrosis measured as expression of collagen I and III (*Col1a2* and *Col3a1* (**D-E**, respectively)), in the left ventricle (LV) of wild-type (WT) and *Sdc4-Tg* mice at 6-8 (n=8) and 60 (n=5) weeks of age, normalized to expression of *Gapdh*. Data are mean±SEM. Statistical differences were tested using t-test vs. respective WT control at 6-8w or 60w, respectively, *p<0.05, **p<0.01.


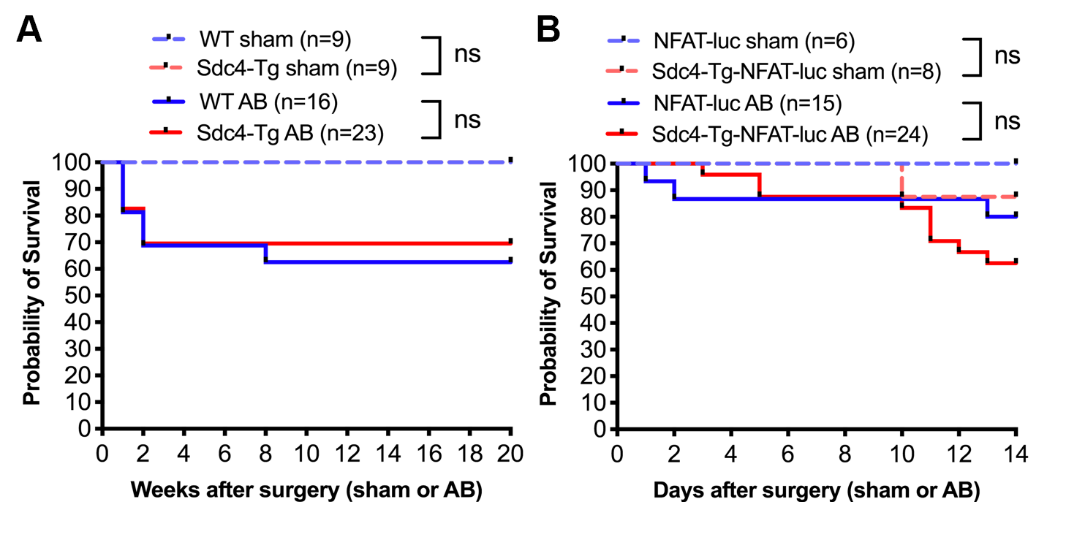


**Fig. S3 *Sdc4-Tg* mice show similar mortality to controls upon pressure overload**

Kaplan-Meier survival curves of *Sdc4-Tg* and WT (**A**) and *Sdc4-Tg-NFAT-luc* and *NFAT-luc* (**B**) mice upon sham operation or pressure overload induced by aortic banding (AB). Survival was monitored over 20 weeks (A) and 14 days (B). ns, non-significant.


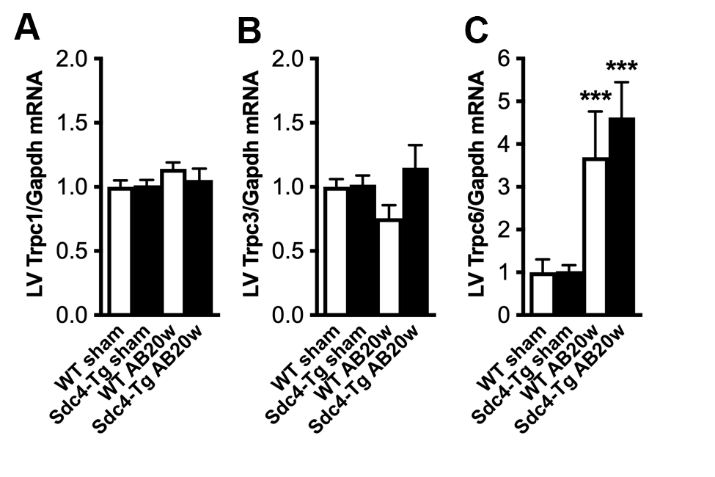


**Fig. S4 *Sdc4-Tg* mice do not show differences in expression of TRPC 1, 3, and 6**

Relative mRNA expression of *Trpc1* (**A**), *Trpc3* (**B**), and *Trpc6* (**C**), encoding transient receptor potential channel (TRPC) 1, 3, and 6 in the left ventricle (LV) of wild-type (WT) and *Sdc4-Tg* mice 20 weeks post-aortic banding (AB), n=7-9. Gene expression was normalized to *Gapdh*. Data are mean±SEM. Statistical differences were tested using one-way ANOVA with Dunnett´s post-testing vs. WT sham, ***p<0.001; and vs. WT AB20w.

**
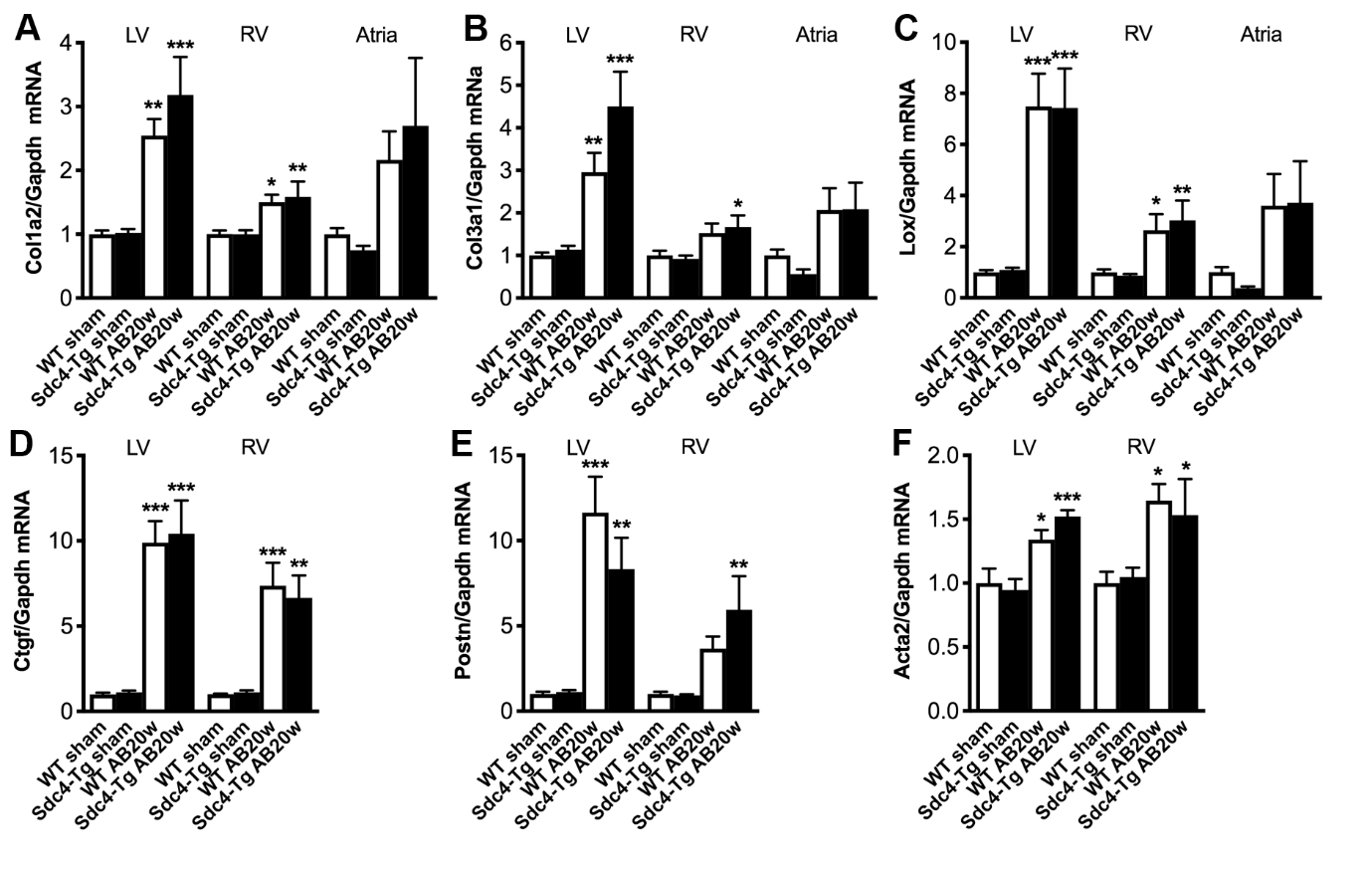
Fig. S5 *Sdc4-Tg* mice do not show exacerbated cardiac fibrosis upon pressure overload**

Relative mRNA expression of central molecules in cardiac fibrosis, *Col1a2* (encoding collagen I; **A**), *Col3a1* (encoding collagen III; **B**), *Lox* (encoding the collagen cross-linking enzyme lysyl oxidase; **C**), *Ctgf* (encoding connective tissue growth factor, **D**), *Postn* (encoding periostin; **E**), *Acta2* (encoding alpha-smooth muscle actin; **F**), in the left ventricle (LV), right ventricle (RV) and atria of wild-type (WT) and *Sdc4-Tg* mice 20 weeks post-aortic banding (AB, n=7-9). Gene expression was normalized to *Gapdh*. Data are mean±SEM. Statistical differences were tested using one-way ANOVA with Dunnett´s post-testing vs. WT sham, *p<0.05, **p<0.001, ***p<0.001; and vs. WT AB20w.

**
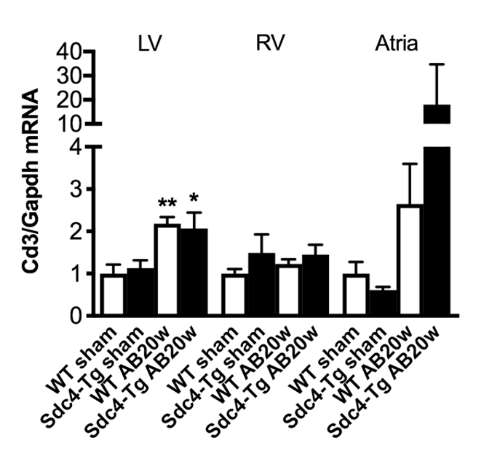
**

**Fig. S6 *Sdc4-Tg* mice do not show exacerbated immune cell infiltration**

Relative mRNA expression of *Cd3* (encoding cluster of differentiation 3, a transmembrane receptor expressed on T-cells)) in the left ventricle (LV), right ventricle (RV) and atria of wild-type (WT) and *Sdc4-Tg* mice 20 weeks post-aortic banding (AB, n=7-9). Gene expression was normalized to *Gapdh*. Data are mean±SEM. Statistical differences were tested using one-way ANOVA with Dunnett´s post-testing vs. WT sham, *p<0.05, **p<0.001; and vs. WT AB20w.

**
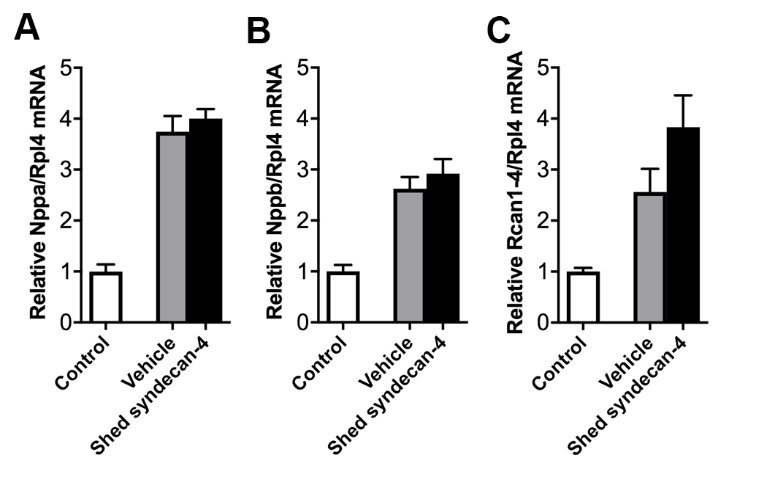
**

**Fig. S7 The shed syndecan-4 ectodomain does not induce NFAT signalling in cultured cardiomyocytes**

Cardiomyocytes from neonatal rats (NCM) were treated with conditioned culture medium from human embryonic kidney (HEK) 293 cells containing shed syndecan-4 ectodomain [3], as shown in Fig. S1. Medium from vehicle-transfected HEK293 cells was used as vehicle control, next to non-treated (control) cells (n=9). Relative mRNA expression of signature molecules of heart failure (*Nppa* and *Nppb* encoding atrial and brain natriuretic peptides; **A-B**, respectively), and NFAT activation (*Rcan1-4*, **C**) in NCM. Gene expression was normalized to *Rpl4*. Data are mean±SEM. Statistical differences were tested using one-way ANOVA with Dunnett´s multiple comparison test vs. vehicle control.

**Supplemental Tables**

**Table SI. Characteristics of *Sdc4-Tg* mice after AB**

|  | **2 weeks** | | | | **6 weeks** | | | | **12 weeks** | | | |
| --- | --- | --- | --- | --- | --- | --- | --- | --- | --- | --- | --- | --- |
|  | **Sham**  **WT** | **Sham**  ***Sdc4-Tg*** | **AB**  **WT** | **AB**  ***Sdc4-Tg*** | **Sham**  **WT** | **Sham**  ***Sdc4-Tg*** | **AB**  **WT** | **AB**  ***Sdc4-Tg*** | **Sham**  **WT** | **Sham *Sdc4-Tg*** | **AB**  **WT** | **AB**  ***Sdc4-Tg*** |
| ***M-mode echocardiography*** | | | | | | | | | | | | |
| **N** | 3 | 4 | 6 | 7 | 3 | 3 | 6 | 8 | 6 | 7 | 8 | 11 |
| **LAD (mm)** | 1.87  ± 0.05 | 1.76  ± 0.06 | 2.47  ± 0.16 | 2.33  ± 0.16 | 1.76  ± 0.06 | 1.70  ± 0.04 | 2.24  ± 0.17 | 2.48*  ± 0.15 | 1.64  ±0.02 | 1.64  ±0.03 | 2.15  ±0.11** | 2.44  ±0.13*** |
| **LVPWd (mm)** | 0.70  ± 0.02 | 0.69  ± 0.01 | 1.06  ± 0.03*** | 0.99  ± 0.03*** | 0.64  ± 0.02 | 0.62  ± 0.01 | 1.05  ± 0.06*** | 1.06  ± 0.03*** | 0.66  ±0.01 | 0.68  ±0.01 | 1.06  ±0.06*** | 1.12  ±0.02*** |
| **IVSd (mm)** | 0.72  ± 0.01 | 0.68  ± 0.02 | 1.03  ± 0.05*** | 1.02  ± 0.03*** | 0.64  ± 0.02 | 0.70  ± 0.02 | 1.06  ± 0.05*** | 1.00  ± 0.03*** | 0.70  ±0.02 | 0.69  ±0.02 | 1.07  ±0.04*** | 1.15  ±0.02*** |
| **LVIDd (mm)** | 4.24  ± 0.29 | 4.16  ± 0.11 | 4.13  ± 0.11 | 3.94  ± 0.15 | 4.22  ± 0.20 | 4.57  ± 0.10 | 4.70  ± 0.18 | 4.36  ± 0.15 | 4.19  ±0.12 | 4.14  ±0.11 | 4.22  ±0.12 | 4.62  ±0.20 |
| **FS (%)** | 26.00  ± 2.65 | 24.75  ± 1.65 | 18.83  ± 2.32 | 24.14  ± 2.02 | 31.67  ± 6.06 | 25.33  ± 2.33 | 17.83  ± 1.85* | 18.00  ± 2.27* | 30.50  ±1.98 | 27.57  ±1.91 | 24.63  ±1.96 | 16.73  ±1.93***,# |
| **Calc. LV mass (mg)** | 115.00  ± 14.66 | 105.40  ± 6.74 | 183.30  ± 13.45** | 160.10  ± 7.93* | 100.40  ± 6.64 | 118.50  ± 6.31 | 233.70  ± 30.00** | 196.40  ± 13.75* | 96.24  ±2.19 | 96.35  ±3.24 | 195.70  ±15.95*** | 247.60  ±19.15***,# |

M-mode echocardiography data (mean±SEM) of male *Sdc4-Tg* and wild-type (WT) littermate control mice two, six and twelve weeks after aortic banding (AB) or sham operation. LAD, left atrial diameter; IVSd, interventricular septum thickness in diastole; LVPWd, left ventricular posterior wall thickness in diastole; LVIDd, left ventricular internal diameter in diastole; FS, fractional shortening. One-way ANOVA with Dunnett´s post-test vs. WT sham; *p<0.05; **p<0.01; ***p<0.001. One-way ANOVA with Dunnett´s post-test vs. WT AB; #p<0.05.

**Table SII. Characteristics of *Sdc4-Tg-NFAT-luc* mice after AB**

|  | **Sham**  ***NFAT-luc*** | **Sham**  ***Sdc4-Tg-NFAT-luc*** | **AB 2w**  ***NFAT-luc*** | **AB 2w**  ***Sdc4-Tg-NFAT-luc*** |
| --- | --- | --- | --- | --- |
| ***Biometric data*** | | | | |
| **N** | 6 | 7 | 12 | 15 |
| **BW (g)** | 29.68  ± 0.63 | 29.96  ± 0.41 | 27.50  ± 0.45 | 26.05  ± 0.60*** |
| **LVW/BW (mg/g)** | 4.87  ± 0.13 | 4.58  ± 0.15 | 7.16  ± 0.25*** | 8.35  ±0.35***, # |
| **LW/BW (mg/g)** | 5.18  ± 0.14 | 5.35  ± 0.19 | 9.67  ± 1.02* | 13.34  ± 1.27***, # |

Body and organ weights (mean±SEM) of male *Sdc4-Tg-NFAT-luciferase* (luc) and *NFAT-luc* control mice after two weeks of aortic banding (AB) or sham operation. BW, body weight; LVW, left ventricular weight; LW, lung weight. One-way ANOVA with Dunnett´s post-test vs. NFAT-luc sham; *p<0.05; ***p<0.001, and NFAT-luc AB; #p<0.05.

**Table SIII. Gene expression assays and antibodies**

| **Gene:** | **Mouse qPCR assays:** | **Rat qPCR assays:** |
| --- | --- | --- |
| *Sdc1* | Mm00448918_m1 |  |
| *Sdc2* | Mm00484718_m1 |  |
| *Sdc3* | Mm01179831_m1 |  |
| *Sdc4* | Mm00488527_m1 | Rn00561900_m1 |
| *Nppa* | Mm01255747_g1 | Rn00664637_g1 |
| *Nppb* | Mm00435304_g1 | Rn00580641_m1 |
| *Rcan1-4* | Mm01213406_m1 | Rn00596606_m1 |
| *Ppp3cb* | Mm00920265_m1 |  |
| *Nfatc4* | Mm00452375_m1 |  |
| *Col1a2* | Mm00483888_m1 |  |
| *Col3a1* | Mm01254476_m1 |  |
| *Lox* | Mm00495386_m1 |  |
| *Ctgf* | Mm01192932_g1 |  |
| *Postn* | Mm01284919_m1 |  |
| *Acta2* | Mm01546133_m1 |  |
| *Cd3* | Mm01179194_m1 |  |
| *Trpc1* | Mm00441975_m1 |  |
| *Trpc3* | Mm00444690_m1 |  |
| *Trpc6* | Mm01176083_m1 |  |
| *Gapdh* | Mm99999915_g1 |  |
| *Rpl4* |  | Rn00821091_g1 |
| **Antibodies for immunoblotting and immunoprecipitation:** | | |
| Syndecan-4 cytoplasmic epitope | custom made [1], Genscript Corporation | |
| Syndecan-4 extracellular epitope | BD550351, clone KY/8.2, BD Biosciences | |
| Heparan sulfate neo-epitope 3G10 | F69-3G10, 370260-1, Amsbio | |
| Calcineurin (PP2B Aβ) | 07-068-I, Sigma Aldrich | |
| Vinculin | V9131, Sigma Aldrich | |
| Influenza hemagglutinin (HA) | 3724, Cell Signalling | |

Pre-designed assays (Applied Biosystems) used for qPCR analyses, and antibodies used for immunoblotting and immunoprecipitation.

**References**

[1] A.V. Finsen, I.G. Lunde, I. Sjaastad, E.K. Østli, M. Lyngra, H.O. Jarstadmarken, A. Hasic, S. Nygård, S.A. Wilcox-Adelman, P.F. Goetinck, T. Lyberg, B. Skrbic, G. Florholmen, T. Tønnessen, W.E. Louch, S. Djurovic, C.R. Carlson, G. Christensen, Syndecan-4 is essential for development of concentric myocardial hypertrophy via stretch-induced activation of the calcineurin-NFAT pathway, PLoS One 6(12) (2011) e28302.

[2] M.E. Strand, K.M. Herum, Z.A. Rana, B. Skrbic, E.T. Askevold, C.P. Dahl, M. Vistnes, A. Hasic, H. Kvaløy, I. Sjaastad, C.R. Carlson, T. Tønnessen, L. Gullestad, G. Christensen, I.G. Lunde, Innate immune signaling induces expression and shedding of the heparan sulfate proteoglycan syndecan-4 in cardiac fibroblasts and myocytes, affecting inflammation in the pressure-overloaded heart, FEBS J 280(10) (2013) 2228-2247.

[3] M.E. Strand, J.M. Aronsen, B. Braathen, I. Sjaastad, H. Kvaløy, T. Tønnessen, G. Christensen, I.G. Lunde, Shedding of syndecan-4 promotes immune cell recruitment and mitigates cardiac dysfunction after lipopolysaccharide challenge in mice, J Mol Cell Cardiol 88 (2015) 133-144.
